# Iruplinalkib (WX‑0593), a novel ALK/ROS1 inhibitor, overcomes crizotinib resistance in preclinical models for non-small cell lung cancer

**DOI:** 10.1007/s10637-023-01350-x

**Published:** 2023-04-10

**Authors:** Yingying Yang, Qingmei Zheng, Xinmei Wang, Shuyong Zhao, Wenshu Huang, Linchao Jia, Cuicui Ma, Shicong Liu, Yongpeng Zhang, Qianqian Xin, Yan Sun, Shansong Zheng

**Affiliations:** 1 Department of Nonclinical Development, Qilu Pharmaceutical Co., Ltd., Jinan, 250104 China; 2 Department of Clinical Development, Qilu Pharmaceutical Co., Ltd., Jinan, 250104 China; 3 Department of Clinical Pharmacology, Qilu Pharmaceutical Co., Ltd., 8888 Lvyou Road, High-tech Zone, Jinan, 250104 China

**Keywords:** Iruplinalkib, Anaplastic lymphoma kinase, Tyrosine kinase inhibitors, Crizotinib resistance, Resistance mechanisms, Non-small cell lung cancer

## Abstract

Despite remarkable initial responses of anaplastic lymphoma kinase (ALK) inhibitors in *ALK*-positive non-small cell lung cancer (NSCLC) patients, cancers eventually develop resistance within one to two years. This study aimed to compare the properties of iruplinalkib (WX‑0593) with other ALK inhibitors and report the comprehensive characterization of iruplinalkib against the crizotinib resistance. The inhibitory effect of iruplinalkib on kinase activity was detected. A kinase screen was performed to evaluate the selectivity of iruplinalkib. The effect of iruplinalkib on related signal transduction pathways of ALK and c-ros oncogene 1 (ROS1) kinases was examined. The cellular and in vivo activities of ALK inhibitors were compared in engineered cancer-derived cell lines and in mice xenograft models, respectively. Human hepatocytes derived from three donors were used for evaluating hepatic enzyme inducing activity. HEK293 cell lines expressing transportors were used to invesigated the drug interaction potential mediated by several transporters. The results showed iruplinalkib potently inhibited the tyrosine autophosphorylation of wild-type ALK, ALK^L1196M^, ALK^C1156Y^ and epidermal growth factor receptor (EGFR)^L858R/T790M^. The inhibitory effects of iruplinalkib in patient-derived xenograft and cell line-derived xenograft models were observed. Moreover, iruplinalkib showed robust antitumor effects in BALB/c nude mice xenograft models with ALK-/ROS1-positive tumors implanted subcutaneously, and the tumor suppressive effects in crizotinib-resistant model was significantly better than that of brigatinib. Iruplinalkib did not induce CYP1A2, CYP2B6 and CYP3A4 at therapeutic concentration, and was also a strong inhibitor of MATE1 and MATE2K transporters, as well as P-gp and BCRP. In conclusion, iruplinalkib, a highly active and selective ALK/ROS1 inhibitor, exhibited strong antitumor effects in vitro and in crizotinib-resistant models.

**Supplementary Information:**

The online version contains supplementary material available at 10.1007/s10637-023-01350-x.

## Introduction

Incidence and mortality of lung cancer are progressively increasing worldwide [1], and non-small cell lung cancer (NSCLC) is the most common histological type, which accounts for 80–85% of lung cancer [2–4]. Oncogenic driver fusions caused by chromosomal rearrangement, such as echinoderm microtubule-associated protein-like 4-anaplastic lymphoma kinase (*EML4-ALK*) and CD74-c-ros oncogene 1 (*CD74-ROS1*), are known to play important roles in tumorigenesis and progression in NSCLC [5]. Due to its high activity in lung cancers with *ALK* or *ROS1* chromosomal rearrangements, crizotinib, the first-generation ALK tyrosine kinase inhibitor (TKI), is now widely used in clincal practice [6–9].

Data from preclinical studies suggest that crizotinib can inhibit tyrosine phosphorylation of activated ALK and ROS1 at nanomolar concentrations [10–12]; nonetheless, most patients invariably acquire resistance to crizotinib within 1–2 years of treatment, with relapse and progression in central nervous system being particularly common [13–15]. Multiple second- (e.g., ceritinib, alectinib, and brigatinib) and third-generation (e.g., loratinib) ALK-TKIs emerged with higher potency and greater central nervous system penetration than crizotinib [16–18]. However, disease progression occurs in treated patients even after a period of treatment due to the phenomenon of acquired resistance, which may be caused by several known or unknown mechanisms. The main mechanisms of resistance identified so far include *ALK* gene mutations, amplification [13, 14, 19–21], and activation of other signaling pathways [14, 20, 22].

Due to the inconsistent range of activity of each ALK-TKI, the variability of *ALK* kinase domain resistance mutation sites is also large. *ALK* kinase domain resistance mutations are currently confirmed in approximately one-third of crizotinib-refractory tumors, including L1196M, L1152R, G1202R, G1269A, 1151Tins, S1206Y, C1156Y, F1174C, and D1203N [20, 23]. In contrast, second-generation ALK inhibitors, exemplified by ceritinib, were found to be ~ 20-fold more potent against ALK than crizotinib in an in vitro enzymatic study, with a half-maximal growth inhibition concentration (GI_50_) value of 0.15 nM, and the growth of Ba/F3 cells expressing *ALK* harboring L1196M, G1269A, I1171T, and S1206Y mutations was effectively inhibited by ceritinib, but ceritinib has no effect on the G1202R and F1174C mutations in *ALK* [24]; Brigatinib has pan-ALK inhibitory activity, especially ALK^G1202R^, but it has weak effects on epidermal growth factor receptor (EGFR)^L858R^ and EGFR^T790M^, and has no effect on crizotinib resistance associated with ROS1^G2032R^ [25]. There is still insurmountable resistance in therapy, making these TKIs much less effective, so the exploration of resistance mechanisms is beneficial in providing new options for subsequent treatment of patients. Drug resistance remains the most pressing issue to improve the survival time of patients with advanced-stage NSCLC.

It is reported that the downstream signaling pathways affected by the EML4-ALK fusion protein include the RAS-RAF-MEK-ERK pathway (i.e., the MAPK/ERK pathway) that regulates cell proliferation, and the PI3K-AKT-mTOR pathway involved in cell survival and JAK-STAT pathway [26]. Therefore, the effect of iruplinalkib (WX‑0593) on these signaling pathways molecules during incubation was tested in this study.

The family of solute carriers (SLC) as a site of drug-drug interactions (DDIs) now has been recognized as playing an important role in the pharmacokinetic and pharmacodynamic profile of lots of drugs by controlling absorption, distribution, and elimination processes [27]. Most of these DDIs can be directly associated with the action of a select group of exogenous transport proteins such as OATP, OAT, OCT and MATE, the ATP binding cassette (ABC) and the SLC superfamily of membrane transport proteins [28] which are recognized as important determinants of the absorption, distribution, metabolism and excretion of many xenobiotic compounds [29, 30]. Both the US Food and Drug Administration (FDA) and European Medicines Agency (EMA) guidelines suggested that new investigational drugs are required to be evaluated in the early stages of development for the selection of substrates or inhibitors of transporter proteins to predict DDI liability [31–33].

Iruplinalkib is a novel ALK/ROS1 inhibitor designed to inhibit ALK kinase activity in wild-type (WT) and ALK inhibitor-resistant mutations of different fusion types, while effectively inhibiting the activity of different fusion types of ROS1 kinase. The safety and activity of iruplinalkib in *ALK-* or *ROS1-*positive NSCLC patients were evaluated in a phase I clinical trial (NCT03389815) [34]. In preclinical studies, iruplinalkib showed great antitumor efficacy in vivo and vitro. Here, we provide the first comprehensive characterization of the mechanism, toxicity, DDIs and drug transporters of iruplinalkib against the resistance of crizotinib.

## Materials and methods

### Compound

Iruplinalkib was provided by Qilu Pharmaceutical Co., Ltd. (Jinan, People’s Republic of China); Brigatinib (AP26113) was obtained from WuXi AppTec; Crizotinib was supplied by HaoYuan Chemexpress, ShangHai Yunli Economic and Trade Co., Ltd. (Purchasing Anrun Medicine); Ceritinib (LDK378) was purchased from Selleckchem/Shanghai WuXi AppTec. Chemical structures of all TKIs were confirmed by standard techniques (liquid chromatography-mass spectrometry and nuclear magnetic resonance). In all tumor xenograft models in this study, target compounds were administered orally at 5 to 10 mL/kg using a sterile 20G injection needle (Popper & Sons, Inc., NY, USA) for infusion.

### Patient-derived xenograft (PDX) and cell line-derived xenograft (CDX) models, and SD rats

Female BALB/c nude mice (4–6 weeks old) were provided by Shanghai Sipple-Bikai Laboratory Animal Co., Ltd. and Beijing Vital River Laboratory Animal Technology Co., Ltd. The mice were housed in a specific pathogen-free environment under a 12 h light/dark cycle, and had free access to sterile animal feed and water.

PDX and CDX models were used for in vivo studies. Patient-derived tumors tissues were divided into about 30 mm^3^ and transplanted into the right dorsal subcutis. Treatment began when the tumor size reached to 200 mm^3^. The mice were randomly assignied into the corresponding dosing groups (9 mice in each group). Drugs were deliverd via daily oral for 4 weeks. Tumor size was measured periodically using a digital caliper (Cal Pro, Sylvac, Switzerland), and tumor volume was calculated as 0.5 × length × width^2^. For more detailed information, please refer to Supplementary materials. All procedures and protocols of xenograft models were approved by the Institutional Animal Care and Use Committee of WuXi AppTec.

20 males and females SD rats (*Rattus norvegicus*) respectively, aged about 7 weeks, with body weight range of 192.25–233.05 g for females and 225.14–258.47 g for males, were used. Females were infertile. The rats were housed in 2323 A under a 12 h light/dark cycle, with the humidity of 51.5–67.5%, temperature of 21.6–23.4 °C and free access to sterile animal feed and water.

All animal projects were implemented following the Guidelines for Research on the Care and Use of Laboratory Animals of the Institute for Laboratory Animal Research and the Public Health Service Policy on Human Care of Laboratory Animals. Experiments were approved by Institutional Animal Care and Use Committee (IACUC).

### Cell lines and cell culture

Karpas 299, NCI-H1975, NCI-H3122, Ba/F3, HCC-78, NIH-3T3, Caco-2, and HEK293 cells were used in this study. The engineered Karpas 299 (*NPM-ALK* fusion gene) cell line was obtained from Sigma-Aldrich. NCI-H1975 (*EGFR*^L858R/T790M^ mutation) was obtained from ATCC. The engineered NIH-3T3 (*CD74-ROS1* fusion gene) cells was obtained from Nanjing GenScript Biotechnology Co., Ltd. The engineered Ba/F3 (*SLC34A2-ROS1* fusion gene) cell line was obtained from Kyinno Biotechnology (Beijing) Co., Ltd. The HCC-78 (*SLC34A2*-*ROS1* fusion gene) cell line was purchased from Shanghai Hongshun Biotechnology Co., Ltd. The engineered NIH-3T3 (CD74-ROS1 fusion gene) cells was obtained from Nanjing GenScript Biotechnology Co., Ltd. The engineered HEK293 cell lines with *OATP1B1* (Lot. No ESH6G27), *OATP1B3* (Lot. No ESH7G27), *OCT2* (Lot. No ESH9G27), *OAT1* (Lot. No ESHCG27), *OAT3* (Lot. No ESHAG27), *MATE1* (Lot. No ESH8G27), and *MATE2K* (Lot. No ESHDG27) were purchased from GenoMembrane and validated using the specific substrate for each transporter. All cells were cultured at 37℃ in a 5% CO_2_ atmosphere. Cell culture reagents were obtained from Life Technologies, Inc. See Supplemental Table 1 for details on the source of the cell lines and the media used. The detection of engineered cell lines above were shown in Supplemental Fig. 1.

### Cell proliferation analysis

Cell proliferation assay was performed using methods reported in the previous article. Cell proliferation was measured using the CellTiter-Glo^®^ (CTG) Luminescent Cell Viability Assay (Promega, WI, USA). Cells were seeded in 96-well plates at 15,000 cells per well in detection medium containing 0.5% fetal bovine serum and placed in an incubator at 37℃ and 5% CO_2_ overnight. On the following day, iruplinalkib was serially diluted with DMSO, further diluted in assay medium, and added to the designated wells for serial dilution of iruplinalkib to the target gradient concentration. After the cells were incubated for 24 h, the number of viable cells at each concentration was determined by CTG assay, and the half-maximal inhibition concentration (IC_50_) values were calculated by concentration-response curve fitting utilizing a four-parameter logistic regression model.

### Kinase activity and selectivity assay

IC_50_ values for the inhibition of iruplinalkib on ALK^WT^, ALK^L1196M^, ALK^C1156Y^, and EGFR^L858R/T790M^ mutant kinases were detected by HTRF^®^ (Cisbio, MA, USA). After the kinase proteins, Ulight-peptide species, adenosine triphosphate (ATP), and compound were mixed, the reaction was carried out at room temperature. Reactions were quenched by the addition of ethylene diamine tetraacetic acid buffer containing Eu antibody and XL665-streptavidin at the indicated time points. Plate reading was performed using an Envision (Perkin Elmer, MA, USA) multifunctional microplate reader. The ratio of 665 nm/620 nm reflects the degree of phosphorylation.

The selectivity of iruplinalkib against 27 human kinases was examined by Z’-LYTE^®^ technology (Invitrogen, MA, USA): kinase proteins, peptides, ATP, and compounds were mixed and reacted at room temperature, and at indicated time points, added developing solution, and the degree of inhibition of kinase phosphorylation was detected using the ratio of 445 nm/520 nm.

### Transfection

The gene of *EML4-ALK*^WT^, *EML4-ALK*^L1196M^, *EML4-ALK*^C1156Y^ were synthesized using confidential methods, and introduced into Ba/F3 cells. Transfection was performed when cell confluent reached 70–90% in 12-well plates. Opti-MEM medium was used to dilute Lipofectamine 3000 reagent (ThermoFisher, MA, USA) and siRNA separately and mixed well. The mixture was added to the cells after incubation at room temperature for 15 min, then at 37℃ for 2–4 days. The results of transfection after 24 h were comfirmed to ensure successful transfection (Supplementary Fig. 1a). The engineered NIH-3T3 cell line and Ba/F3 cell line were also detected by western blot (Supplementary Fig. 1b-c).

### Western blot

In general, the cells were exposed to the drug and were harvested for western blot analysis. SDS-PAGE was used to separate the proteins of interest, which were then transferred to a nitrocellulose (NC) filtration membrane and soaked in a blocking solution [5% non-fat dry milk in tris-buffered saline with Tween 20 (TBST)] for 1 h at room temperature. Thereafter, the membrane was probed overnight with primary antibodies at 4℃ and washed three times with TBST. The corresponding secondary antibody diluent was added, incubated at room temperature for 1 h, and washed 3 times with TBST. After adding the enhanced chemiluminescence developer to the NC filtration membrane, it is developed in the imaging system (ChemiDoc™ XRS^+^, BIO-RAD). See Supplementary Table 2 for details of the antibodies used.

### Statistical analysis

Kinase activity and cell proliferation IC_50_ results were analyzed by IDBS’ XLfit5 (IDBS, UK) or GraphPad Prism (GraphPad Software, Inc., CA, USA). In western blot experiments, Image Lab software (Bio-Rad, CA, USA) was used to quantify the density intensity of immunoblot chemiluminescence bands. Data are presented as means ± standard deviation. Statistical analysis was performed in Graphpad Prism using one-way ANOVA test. Significance was established for *P*-values < 0.05.

## Results

### Iruplinalkib displayed inhibitory effects in vitro in ALK-/ROS-positive cell lines

**Fig. 1:**
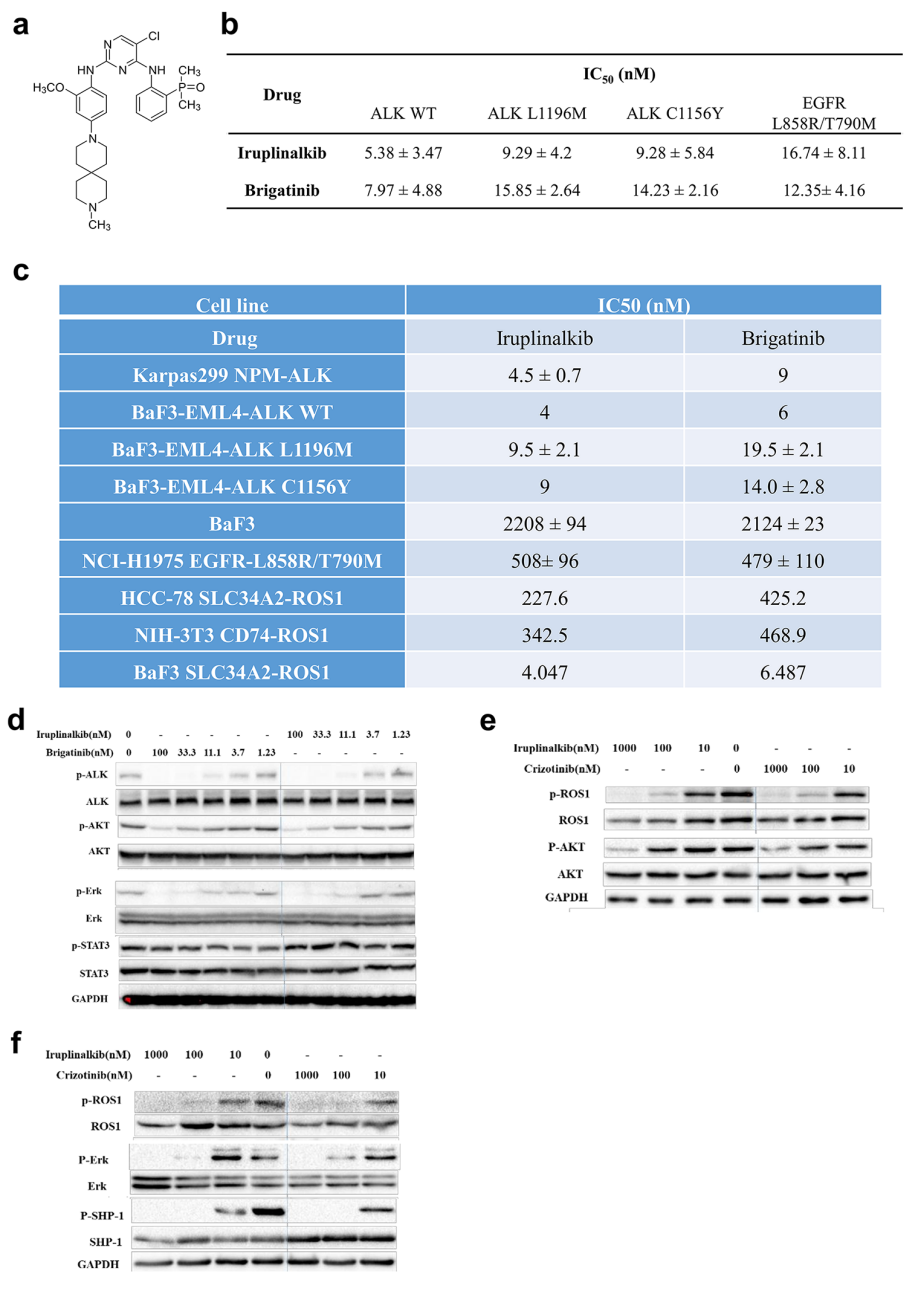
Iruplinalkib inhibited kinase activity and the downstream signaling pathways of ALK and ROS1 in vitro. (a) Chemical formula of iruplinalkib. (b) Iruplinalkib and brigatinib inhibited the kinase activity of ALK^WT^, ALK^L1196M^, ALK^C1156Y^, and EGFR^L858R/T790M^. (c) The kinase selectivity of iruplinalkib and brigatinib to the fusion protein. Proliferation of Karpas299 (*NPM-ALK*), Ba/F3, Ba/F3 (*EML4-ALK*^WT^), Ba/F3 (*EML4-ALK*^L1196M^), Ba/F3 (*EML4-ALK*^C1156Y^), NCI-H1975 (*EGFR*^L858R/T790M^), HCC-78 (*SLC34A2-ROS1*), NIH-3T3 (*CD74-ROS1*), and Ba/F3 (*SLC34A2-ROS1*) cells. (d) The inhibitory effects of iruplinalkib on the phosphorylation of ALK, PI3K/AKT, RAS/MAPK and JAK/STAT3 pathways in NCI-H3122 cells, respectively. (e) Iruplinalkib inhibited phosphorylation of ROS1, PI3K/AKT and RAS/MAPK pathways in NIH-3T3 cells stably expressing *CD74-ROS1*, respectively. (f) Iruplinalkib inhibited phosphorylation of ROS1, RAS/MAPK and SHP-1 pathways in Ba/F3 cells stably expressing *SLC34A2-ROS1*, respectively

Iruplinalkib was designed to overcome resistance mutations identified in the clinic, especially ALK and ROS1. There was no chiral center in the iruplinalkib structure (Fig. 1a). In kinase assays, iruplinalkib inhibited the total tyrosine autophosphorylation of ALK^WT^, ALK^L1196M^, ALK^C1156Y^ and EGFR^L858R/T790M^ mutant with IC_50_ values in the range of 5.38–16.74 nM, and showed similar inhibitory potencies with brigatinib (Fig. 1b).

The selectivity of iruplinalkib against a number of non-ALK kinases was further evaluated. The results showed that iruplinalkib potently inhibited WT EGFR (IC_50_, 35 nM), but did not inhibit other 26 kinases strongly (Supplementary Table 3).

The activity of iruplinalkib on cell viability was next examined in several cell lines. In models harboring WT ALK fusion proteins [Karpas 299 (*NPM-ALK*) and Ba/F3 (*EML4-ALK*^WT^)], iruplinalkib exhibited similar cell growth inhibitory potency compared with brigatinib (IC_50_, 4–9 nM). However, in Ba/F3 cells expressing EML4-ALK^L1196M^ or EML4-ALK^C1156Y^ fusion proteins, iruplinalkib was slightly more potent. Cell growth IC_50_ values for iruplinalkib were 9.5 and 9 nM, compared with 19.5 and 14 nM, respectively, for brigatinib.

In Ba/F3 cells expressing WT SLC34A2-ROS1 fusion protein, iruplinalkib had comparable activity with brigatinib (IC_50_, 4.074 and 6.487 nM, respectively). However, HCC-78 cells expressing WT SLC34A2-ROS1 fusion protein, NCI-H1975 cells with EGFR^L858R/T790M^ and NIH-3T3 cells with CD74-ROS1 expressed rendered resistance to iruplinalkib and brigatinib (IC_50_, 227.6–508 nM; Fig. 1c). Overall, iruplinalkib was a potent ALK/ROS1 inhibitor in cellular assays, and selectively inhibitory to engineered cell lines with *ALK* or *ROS1* rearrangements.

### Iruplinalkib inhibited the phosphorylation of downstream signaling molecules

Results showed that, after 2 h incubation with iruplinalkib, the phosphorylation levels of ALK, AKT, ERK but not STAT3 were reduced, and the raitos of phosphorylated protein to total protein were decreased in a dose-dependent manner, which was similar to brigatinib (Fig. 1d). It suggested that iruplinalkib inhibited the growth of NCI-H3122 cells by inhibiting the phosphorylation of ALK and RAS/MAPK/ERK, PI3K/AKT but not JAK/STAT3 pathways.

It is reported that ROS1-mediated cell signaling pathways include RAS-RAF-MEK-ERK, PI3K-AKT-mTOR and JAK-STAT3 pathways [35]. According to reports and the differences in the expression of signaling pathway proteins in different cells, the key signaling proteins in different signaling pathways were selected for research. Results showed that, after 2 h incubation with iruplinalkib, the phosphorylation levels of ROS1, AKT, ERK in NIH-3T3 (*CD74-ROS1*) cells and ROS1, ERK, SHP-1 in Ba/F3 (*SLC34A2-ROS1*) cells were inhibited, and the raitos of phosphorylated protein to total protein were dose-dependent decreased, which was similar to crizotinib (Fig. 1e). It suggested that iruplinalkib also inhibited of ROS1 and RAS/MEK/ERK, PI3K/AKT and SHP-1 pathways (Fig. 1f).

### Iruplinalkib exhibits robust antitumor effects in xenograft models with ALK fusion and ROS1 fusion

**Fig. 2:**
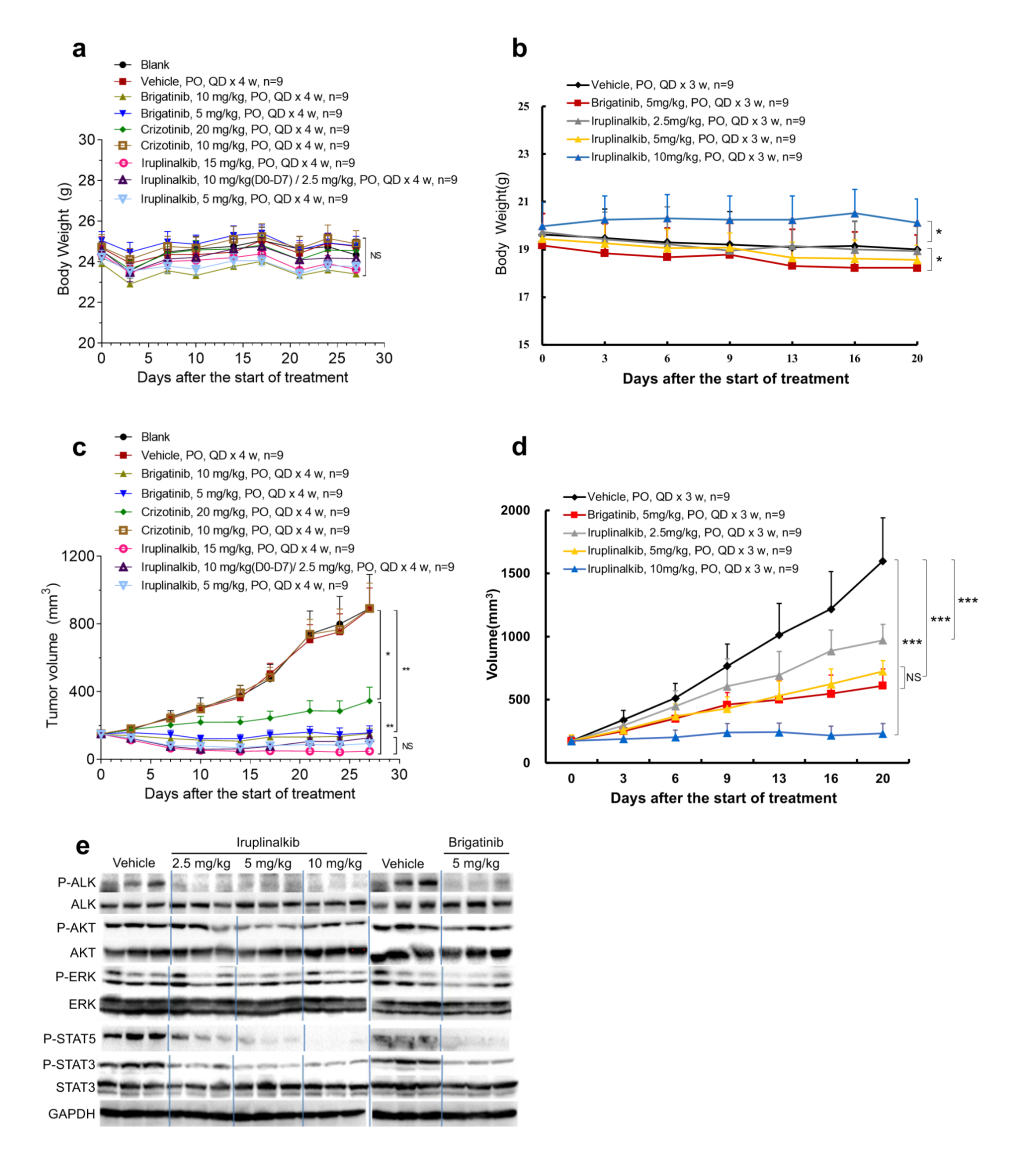
Body weight and antitumor effect of iruplinalkib on BALB/c nude mouse xenograft model and the phosphorylation level of downstream signal molecules in vivo. (a) Body weight and relative body weight change of patient-derived tumor xenograft model LU-01-0015. (b) Body weight change of *EML4-ALK* transfected NCI-H3122 cells subcutaneously transplanted tumor mode; Antitumor effect of iruplinalkib on BALB/c nude mouse xenograft model. (c) LU-01-0015 (*HIP1-ALK*) tumor model; (d) *EML4-ALK* transfected NCI-H3122 cells subcutaneously transplanted tumor model. (e) The phosphorylation level of ALK, AKT, ERK, STAT3 and STAT5 in the tumor tissue in the NCI-H3122 model 8 h after the administration. NS (not significant): P ≥ 0.05. *: P < 0.05. **: P < 0.01. ***: P < 0.001

Since *ALK* fusion is a important oncogenic driver fusion in NSCLC, we tested the antitumor effects of iruplinalkib and other TKIs on different xenograft models with different fusion types of ALK kinase. The LU-01-0015 (*HIP-ALK*) and NCI-H3122 xenograft tumor models were used, respectively. Iruplinalkib, crizotinib, and brigatinib had no effect on the body weight of different nude mouse xenograft models (all P ≥ 0.05, Fig. 2a-b). As we expected, iruplinalkib, brigatinib, crizotinib, and ceritinib were all able to inhibit tumor growth (all P < 0.05). Iruplinalkib exhibited a dose-dependent effect and was similar to brigatinib (at the same dose) in all two models, and all were superior to crizotinib (Fig. 2c-d; see Supplementary Tables 4–5 for details).

### Iruplinalkib exerted antitumor effects by inhibiting ALK phosphorylation and JAK/STAT pathway in vivo

In order to check how iruplinalkib exerts its anti-tumor effects in vivo, tumor tissue samples were collected at 1 and 8 h after the last dose, and the phosphorylation level of downstream signaling pathways molecules were detected. Take NCI-H3122 xenograft model as example, results at 8 h after administration showed that iruplinalkib and brigatinib had similar inhibitory effects on the phosphorylation of ALK, AKT and ERK, which were similar to the in vitro data (Fig. 2e). Interestingly, unlike in the in vitro model, the levels of phosphorylation of STAT3 and STAT5 were found decreased after treating with both iruplinalkib and brigatinib (Fig. 2e). A smaller inhibition of phosphorylation was obersvered at 1 h after administration (Supplementary Fig. 1). The above data suggested that iruplinalkib may inhibit tumor growth by inhibiting ALK phosphorylation and JAK/STAT pathway in NCI-H3122 nude mouse xenograft model.

### Plasma protein binding rate

**Fig. 3:**
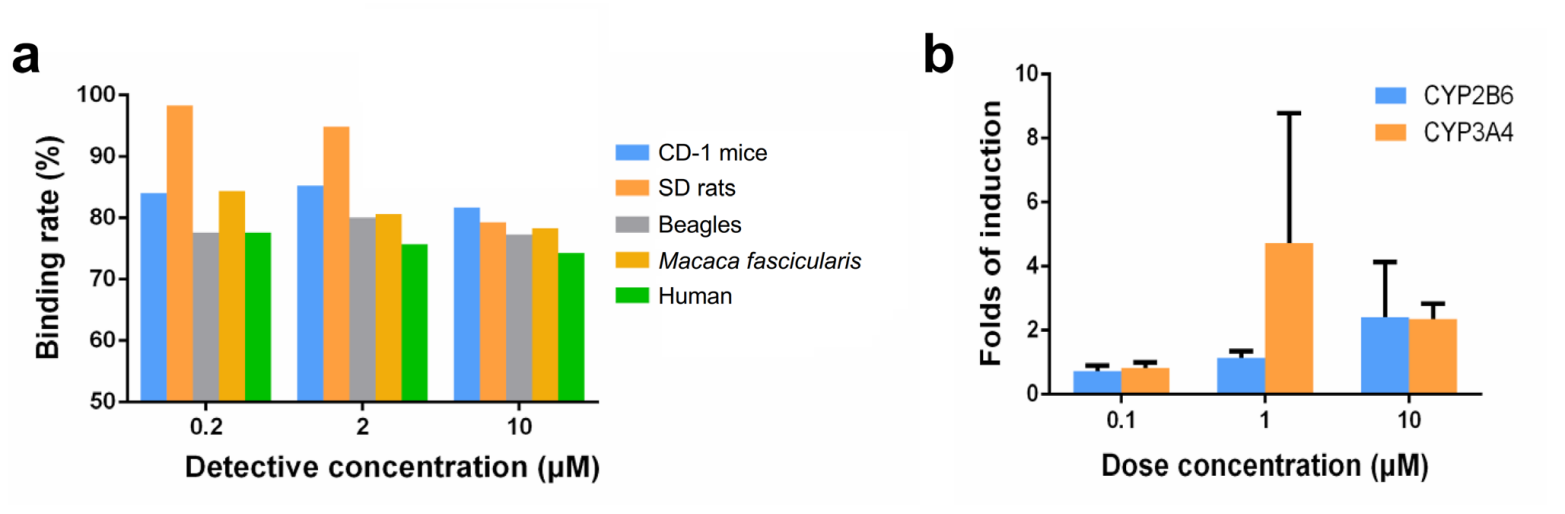
(a) The plasma protein binding rate of iruplinalkib in CD-1 mice, SD rats, Beagles, *Macaca fascicularis* and human. (b) Induction of metabolic enzymes by iruplinalkib

At three test concentrations (0.2, 2 and 10 µM), the plasma protein binding rate of iruplinalkib is as follows (Fig. 3a). The recovery rates of iruplinalkib in the dialysis device were 81.8–99.9%, and the remaining rates in plasma after incubation for 5 h were 95–109.3%, indicating that the compound is stable in plasma during dialysis. Besides, iruplinalkib showed high protein binding rate at 0.2 µM in plasma of SD rats and showed medium protein binding rate at 0.2, 2 and 10 µM in plasma of CD-1 mice, SD rats, Beagle dogs, *Macaca fascicularis* and human. With the increase of concentration, the protein binding rates of iruplinalkib in rat plasma decreased gradually, showing a certain concentration dependence, and there was no concentration dependence of protein binding rate observed in other species plasma.

### Drug-drug interactions for iruplinalkib

Human hepatocytes derived from three donors were used for evaluating for hepatic enzyme inducing activity. The in vitro results showed that iruplinalkib was not an inducer of CYP1A2, CYP2B6, and CYP3A4 at 10 µM, and the cytochrome P450 isoenzyme CYP1A2 has not been induced. The gene expression related to cytochrome P450 isoenzyme CYP3A4 in hepatocytes of one of the three donors increased with iruplinalkib 1 µM. When concentration increased to 10 µM, it did not significantly induced *CYP2B6* gene expression. In donor 1,  the folds of expression were 0.798, 1.36, and 2.62 times, respectively at 0.1, 1, and 10 µM; In donor 2, the folds were 0.862, 0.926, and 4.02 (> 4), respectively; In donor 3, those were 0.526, 1.13, and 0.602, respectively. The induction multiples of *CYP3A4* gene expression in donor 1 were 0.621, 2.99, and 2.38 times, respectively at 0.1, 1 and 10 µM; In donor 2, those were 0.879, 1.80, and 2.81, respectively; In donor 3, those were 0.964, 9.36 (> 4), and 1.86, respectively (Fig. 3b).

### Drug transporters for iruplinalkib

The results of interaction studies with SLC transporters suggested that iruplinalkib can inhibit the uptake activity of the transporters OATP1B1 and OATP1B3. Although iruplinalkib showed a high effective inhibition concentration, it also had obvious dose-dependent characteristics, no substrate activity was found (see Supplemental Tables 6–7). Iruplinalkib had some interference effect on the uptake of OAT1, but no dose-dependent relationship, so it can not be its inhibitor (Supplemental Tables 8–9). Unlike the transporter MATE1, iruplinalkib can effectively inhibits its uptake activity, and this effect was dose-dependent (Supplemental Table 10). Using a stably transfected cell line of HEK293 expressing transporter OATP1B1, OATP1B3 or OCT2, formal substrate investigation tests were performed for three concentration gradients (30 µM, 3 µM, 0.3 µM) of iruplinalkib. The results indicated that the uptake transport activity ratios of the three relevant transporters for this compound were all less than two, and showed non-dose dependent characteristics (Supplemental Table 11). The test results indicated that iruplinalkib was not an uptake substrate for OATP1B1, OATP1B3, OCT2, OAT1 and OAT3.

**Fig. 4:**
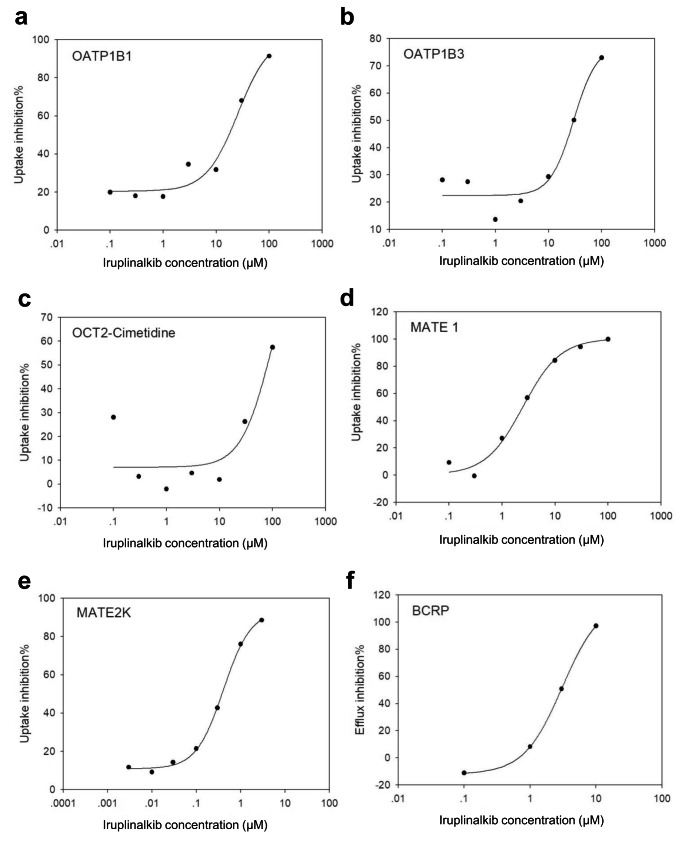
(a-e) Inhibition of OATP1B1-293, OATP1B3-293, OCT2-293, MATE1-293, and MATE2K-293 uptake activity by iruplinalkib; (f) Inhibition of BCRP efflux activity by iruplinalkib on Caco-2 cells

The evaluation of the inhibitory effect of iruplinalkib on the uptake of transporter OATP1B1 and OATP1B3 showed that this inhibitory effect was real, despite its relatively high effective inhibitory concentration. It should be noted that even at low concentrations, iruplinalkib still has a certain inhibitory or interference effect when atorvastatin was used as a substrate (Fig. 4a-b). The inhibitory effect of iruplinalkib on transporter OCT2 uptake below 10 µM was an interference rather than an inhibitory effect (Fig. 4c). And with metformin as the substrate, this case cannot be fitted with a suitable inhibition curve, indicating that iruplinalkib had only a weak inhibitory effect on OCT. The results also showed that iruplinalkib was a stronger inhibitor of transporter MATE1 and MATE2K (Fig. 4d-e).

In vitro studies revealed that iruplinalkib is a transported substrate of P-glycoprotein (P-gp or ABCB1) not for breast cancerresistance protein (BCRP). The ABC efflux transporter inhibitor test was carried out with Caco-2 cell line as the model, which showed that iruplinalkib was a BCRP inhibitor (Table 1). Using 10 µM E3S as the substrate, iruplinalkib significantly inhibited its efflux, and the overall efflux ratio gradually decreased with increasing concentration, showing a dose-dependent characteristic. The results of the fitted inhibition curves are shown (Fig. 4f). Digoxin at 5 µM was used as a substrate, and it showed mostly non-dose-dependent and somewhat disruptive effects on P-gp efflux below 3 µM; at the concentration points of 10 µM and 30 µM, it showed effective with 89.8% and 98.7%, respectively (Table 1). The experimental results showed that iruplinalkib was not an efflux inhibitor of BSEP and MRP2, nor did it have a dose-dependent profile (supplementary Tables 12–13).

**Table 1:** The results of iruplinalkib as P-gp and BCRP substrate, BCRP and P-gp inhibitor investigation

Potential ABC transporters on Caco-2 cells	Reference inhibitors		Standard or examination substrate		Exhaust Ratio			Exhaust inhibition rate (%)
	Compound	Conc (µM)	Compound	Conc (µM)	Mean	SD	CV (%)	
P-gp			Digoxin	5	161.2	42.98	26.7	
	Elacridar	10			0.688	0.220	32.0	99.6
	Verapamil	50			3.349	0.041	1.24	97.9
BCRP			E3S	10	20.54	5.218	25.4	
	Elacridar	10			1.715	0.253	14.7	91.7
	Ko143	10			2.816	0.599	21.3	86.3
P-gp/BCRP/MRP2			Iruplinalkib	0.5	20.34	4.965	24.4	
	Elacridar	10			2.808	0.261	9.29	86.2
	Verapamil	50			3.456	0.659	19.1	83.0
	Ko143	10			20.95	4.181	20.0	-3.0
				5	84.23	12.71	15.1	
	Elacridar	10			0.605	0.197	32.5	99.3
	Verapamil	50			2.626	0.165	6.28	96.9
	Ko143	10			26.14	6.666	25.5	69.0
				50	8.020	1.399	17.4	
	Elacridar	10			0.614	0.130	21.2	92.3
	Verapamil	50			1.954	0.107	5.49	75.6
	Ko143	10			9.889	3.193	32.3	-23.3
BCRP			E3S	10	20.54	5.218	25.4	
	Elacridar	10			1.715	0.253	14.7	91.6
	Ko143	10			2.816	0.599	21.3	86.3
	Iruplinalkib	30			15.13	3.542	23.4	26.3
		10			0.570	0.024	4.14	97.2
		3			10.09	1.418	14.1	50.9
		1			18.84	4.401	23.4	8.3
		0.3			12.79	2.246	17.6	37.7
		0.1			22.82	3.758	16.5	-11.1
P-gp			Digoxin	5	161.2	42.98	26.7	
	Elacridar	10			0.688	0.220	32.0	99.6
	Verapamil	10			3.349	0.041	1.24	97.9
	Iruplinalkib	30			2.165	0.479	22.1	98.7
		10			16.44	3.174	19.3	89.8
		3			191.9	33.67	17.5	-19.1
		1			182.4	24.32	13.3	-13.2
		0.3			154.8	6.244	4.03	3.97
		0.1			210.9	29.16	13.8	-30.9

Note: ABC, adenosine triphosphate binding cassette; SD, standard deviation; CV, coefficient of variation

## Discussion

The discovery of the rearrangement of *ALK* and *ROS1* has led the era of precision medicine for advanced non-small cell lung cancer (NSCLC). Research has progressed rapidly and targeted drugs have been developed one after another. However, most patients with tumors with *ALK* or *ROS1* rearrangement can achieve indefinite but not permanent benefits from targeted therapies. Mechanisms of TKI resistance can be divided into “on-target” mechanisms of acquired tyrosine kinase mutations or “off-target” resistance mechanisms mediated by other molecular mutations.

Here we reported preclinical data for iruplinalkib, a novel ATP-competitive ALK/ROS1 inhibitor, the efficacy and safety of which were currently evaluated in patients who have not been treated with *ALK*-positive NSCLC in a phase III clinical trial (NCT04632758). It had excellent inhibitory activity against autophosphorylation of ALK or ROS1 fusion proteins. In biochemical kinase and cellular assays, IC_50_ values of iruplinalkib for phosphorylation inhibition of ALK^WT^, ALK^L1196M^ and ALK^C1156Y^ were all < 10 nM. Similarly, iruplinalkib was a potent ROS1 inhibitor in multiple assays to inhibit proliferation and phosphorylation levels in cells expressing the ROS1 fusion protein. Iruplinalkib showed strong antitumor effects in various xenograft BALB/c nude mouse models.

In vitro and in vivo studies confirmed that iruplinalkib was an ALK/ROS1 inhibitor with better and selective activity. For *ALK*-rearranged advanced NSCLC, second-generation TKIs, were widely accepted as the front-line standard treatments because of improvement in PFS over crizotinib, impressive central nervous system activity, and favorable toxicity profiles [36–38]. Only a minority of *ALK*-positive patients (~ 20%) developed *ALK*-resistant mutations to crizotinib [39]. In contrast, more than half of patients progressing with second-generation ALK inhibitors had *ALK* resistance mutations, which may reflect the higher potency and selectivity of these drugs compared with crizotinib [39]. Furthermore, the secondary ALK kinase spectra were different from the previous TKI mutation detected. For the second-generation ALK-TKIs, ceritinib, alectinib, and brigatinib, *ALK*^G1202R^ was the most common mutation in disease progression, and the more effective drug is only the third-generation ALK-TKI lolatinib [39, 40]. Iruplinalkib had superior inhibitory activity against the *ALK* mutants resistant to other ALK inhibitors at therapeutic concentration when administrated at 180 mg, once daily [the free steady-state average concentration (C_avg,ss_) was 127 nM] [34]. Also, iruplinalkib had inhibitory activity on *ALK*^G1202R^ mutant which is gatekeeper mutation resistant to all other ALK-TKIs except lorlatinib. Therapeutic resistance in *ROS1*-rearranged NSCLC has largely been studied in the context of crizotinib therapy but the resistance pattern has begun to emerge with the recent FDA approval of the type I ROS1-TKIs entrectinib and the National Comprehensive Cancer Network (NCCN) guideline recommendation of lorlatinib [41, 42]. In crizotinib-resistant tumors, secondary mutations in the exon of the *ROS1* kinase domain have been found clinically. The most common was *ROS1*^G2032R^ [41], which led to resistance to not only crizotinib but also other inhibitors (including entrectinib and lorlatinib) [43]. Iruplinalkib had superior inhibitory activity against the *ROS1* wild type and mutants resistant to crizotinib at therapeutic concentration (180 mg, once daily), except for *ROS1*^G2032R^ and *ROS1*^L1951R^ mutations [34]. Iruplinalkib may be a potent therapy for *ROS1*-positive NSCLC patients who are resistant and not tolerated to crizotinib [34].

Iruplinalkib inhibited the activation of ALK or ROS1 kinase by inhibiting the phosphorylation of ALK or ROS1 and preventing the phosphorylation of downstream signaling molecules (such as ERK, STAT5 and AKT) [44]. According to the studies, the main signal pathways activated by ALK kinase were RAS/MAPK, PI3K/AKT, and JAK/STAT, among which crizotinib can inhibit the phosphorylation of ERK and AKT in NCI-H3122 cells and tumor growth in xenograft tumor models [45]. Therefore, ERK in RAS/MAPK pathway, AKT in PI3K/AKT pathway, and STAT3 and STAT5 in JAK/STAT pathway were selected for detection in this experiment. The results showed that 1 h after administration, the phosphorylation of ERK, STAT3 and STAT5 in tumor tissues in each group was not significantly inhibited, possibly due to the short administration time, the inhibition effect was not obvious. After 8 h of administration, ALK phosphorylation was significantly inhibited in each administration group, and the phosphorylation of STAT3 and STAT5 was also inhibited in a dose-dependent manner. Therefore, in nude mice transplanted tumors in NCI-H3122 cells, the main mechanism of action of iruplinalkib may be to inhibit ALK phosphorylation, and then inhibit tumor growth by inhibiting the downstream JAK/STAT pathway. ALK mediates signalling via the RAS-MAPK, PI3K-mTOR, phospholipase Cγ (PLCγ), RAP1, JAK-STAT and JUN pathways. Proteins such as insulin receptor substrate 1 (IRS1), SHC, growth factor receptor binding 2 (GRB2), SHP-2, C3G, CBL, CRKL, and fibroblast factor receptor substrate 2 (FRS2) interacted with and were activated by ALK and phosphorylated [26]. ROS1 protein autophosphorylation occurred in different locations, and ROS1 autophosphorylation occurred. Phosphorylation occurred at intracellular tyrosine residues at Y2274 which is the binding site for the non-receptor tyrosine phosphatases SHP-2 (PTPN6) and SHP-1 (PTPN11). ROS1 upregulated the SHP-1 and SHP-2 and activated the PI3K/AKT/mTOR, JAK/STAT, and MAPK/ERK pathways [46, 47]. This signaling resulted in the aberrant regulation of a number of genes, ultimately driving cell-cycle progression, survival, proliferation, and angiogenesis [48, 49].

In our work, we used HEK293 cell line introduced by *OATP1B1*, *OATP1B3*, *OCT2*, *OAT1*, *OAT3*, *MATE1*, *MATE2K* to demonstrate that iruplinalkib was a strong inhibitor of MATE1 and MATE2K transporters, as well as P-gp and BCRP. In vitro study showed that iruplinalkib was not the uptake substrate of transporters OATP1B1, OATP1B3, OCT2, OAT1, and OAT3, but could inhibit the uptake activity of OATP1B1, OATP1B3, OCT2, MATE1, and MATE2K. Iruplinalkib had no significant inhibitory effect on OAT1, OAT3, MRP2 and BSEP transporters, and it was a suitable substrate for P-gp, not for OATP1B1, OATP1B3, OCT2, OAT1, OAT3, and BCRP transporters. During the late clinical development process, the DDI risk of iruplinalkib in combination with MATE1, MATE2K, P-gp and BCRP substrate drugs should be closely monitored and evaluated to ensure clinical drug safety.

We previously carried out systematic pharmacokinetic studies to evaluate the absorption, distribution, metabolism, and excretion characteristics of this product in preclinical animal species (mice, SD rats, Beagles, and cynomolgus monkeys) and human body. The pharmacokinetic parameters of iruplinalkib were obtained, providing a pharmacokinetic reference for the dose selection of toxicological research and in vivo pharmacodynamic research [44]. The metabolic characteristics of this product in human body were clarified by using human metabolic enzymes, liver microsomes and hepatocytes, which provided a reference for the selection of animal species in toxicology and the development of clinical pharmacokinetics; in vitro studies had demonstrated that iruplinalkib may have potential induction capacity for CYP2B6 and CYP3A4. However, iruplinalkib had moderate plasma protein binding in human plasma and had low activity in vitro studies. Exposures in clinical applications may well below the maximum tested concentration and the risk of induction-related drug-drug interactions was low, which will require more attention in future clinical trials.

In this study, we tested the effects of iruplinalkib on ALK, ROS1, and their downstream signaling pathway molecules. Autophosphorylation of ALK, ROS1, and phosphorylation of ERK, AKT, and SHP-1 were found to be inhibited in vitro and in vivo, except for STAT3. The level of phosphorylation of STAT3 of NCI-H3122 (*EML4-ALK*) was found decreased in vivo (CDX model) but not in vitro. This may be because that the phosphorylation of STAT3 was at a low level in vitro, while the complex tumor microenvironment such as the possible presence of exogenous polyunsaturated fatty acids and exosomes upregulated STAT3 in tumor cells, making the inhibitory effect of iruplinalkib more pronounced [50, 51]. This also reminded us that the role of tumor microenvironment on tumor growth should not be ignored, and only in vitro experiments are totally insufficient.

Iruplinalkib showed strong antitumor efficacy in the xenograft BALB/c nude mouse model, and the tumor inhibition effect in the crizotinib-resistant model was significantly better than that of brigatinib. Iruplinalkib had a broader spectrum of tumor suppressor activity. Moreover, iruplinalkib showed an effect superiority to crizotinib and brigatinib in various models and cells.

## Conclusion

As a novel ALK/ROS1 inhibitor with high activity and selectivity, iruplinalkib exhibited strong antitumor activity in vitro and in vivo, and also had antitumor effects in crizotinib-resistant models. This study provided a partial molecular biological basis for iruplinalkib in the treatment of crizotinib-resistant or non-resistant *ALK*-positive NSCLC or first-line *ALK*- and *ROS1*-positive NSCLC.

## Electronic supplementary material

Below is the link to the electronic supplementary material.

Supplementary Material 1

## Declarations

### Data sharing Statement

All data generated or analysed during this study are included in this published article and its supplementary information files.

## References

[1] Siegel RL et al (2021) *Cancer Statistics*, CA Cancer J Clin, 2021. 71(1): p. 7–33 10.3322/caac.21654 33433946

[2] Arbour KC, Riely GJ (2019). Systemic therapy for locally Advanced and Metastatic Non-Small Cell Lung Cancer: a review. JAMA.

[3] Huang CY (2017). A review on the effects of current chemotherapy drugs and natural agents in treating non-small cell lung cancer. Biomed (Taipei).

[4] Herbst RS, Morgensztern D, Boshoff C (2018). The biology and management of non-small cell lung cancer. Nature.

[5] Suda K, Mitsudomi T (2020). Emerging oncogenic fusions other than ALK, ROS1, RET, and NTRK in NSCLC and the role of fusions as resistance mechanisms to targeted therapy. Transl Lung Cancer Res.

[6] Solomon BJ (2014). First-line crizotinib versus chemotherapy in ALK-positive lung cancer. N Engl J Med.

[7] Kwak EL (2010). Anaplastic lymphoma kinase inhibition in non-small-cell lung cancer. N Engl J Med.

[8] Camidge DR (2012). Activity and safety of crizotinib in patients with ALK-positive non-small-cell lung cancer: updated results from a phase 1 study. Lancet Oncol.

[9] Shaw AT (2013). Crizotinib versus chemotherapy in advanced ALK-positive lung cancer. N Engl J Med.

[10] Christensen JG (2007). Cytoreductive antitumor activity of PF-2341066, a novel inhibitor of anaplastic lymphoma kinase and c-Met, in experimental models of anaplastic large-cell lymphoma. Mol Cancer Ther.

[11] Shaw AT (2014). Crizotinib in ROS1-rearranged non-small-cell lung cancer. N Engl J Med.

[12] Yasuda H (2012). Preclinical rationale for use of the clinically available multitargeted tyrosine kinase inhibitor crizotinib in ROS1-translocated lung cancer. J Thorac Oncol.

[13] Katayama R (2011). Therapeutic strategies to overcome crizotinib resistance in non-small cell lung cancers harboring the fusion oncogene EML4-ALK. Proc Natl Acad Sci U S A.

[14] Katayama R (2012). Mechanisms of acquired crizotinib resistance in ALK-rearranged lung cancers. Sci Transl Med.

[15] Sakamoto H (2021). Characteristics of central nervous system progression in non-small cell lung cancer treated with crizotinib or alectinib. Cancer Rep (Hoboken).

[16] Shaw AT (2017). Ceritinib versus chemotherapy in patients with ALK-rearranged non-small-cell lung cancer previously given chemotherapy and crizotinib (ASCEND-5): a randomised, controlled, open-label, phase 3 trial. Lancet Oncol.

[17] Novello S (2018). Alectinib versus chemotherapy in crizotinib-pretreated anaplastic lymphoma kinase (ALK)-positive non-small-cell lung cancer: results from the phase III ALUR study. Ann Oncol.

[18] Huber RM (2020). Brigatinib in Crizotinib-Refractory ALK + NSCLC: 2-Year follow-up on systemic and intracranial outcomes in the phase 2 ALTA Trial. J Thorac Oncol.

[19] Choi YL (2010). EML4-ALK mutations in lung cancer that confer resistance to ALK inhibitors. N Engl J Med.

[20] Doebele RC (2012). Mechanisms of resistance to crizotinib in patients with ALK gene rearranged non-small cell lung cancer. Clin Cancer Res.

[21] Sasaki T (2011). A novel ALK secondary mutation and EGFR signaling cause resistance to ALK kinase inhibitors. Cancer Res.

[22] Kim S (2013). Heterogeneity of genetic changes associated with acquired crizotinib resistance in ALK-rearranged lung cancer. J Thorac Oncol.

[23] Gainor JF, Shaw AT (2013). Emerging paradigms in the development of resistance to tyrosine kinase inhibitors in lung cancer. J Clin Oncol.

[24] Friboulet L (2014). The ALK inhibitor ceritinib overcomes crizotinib resistance in non-small cell lung cancer. Cancer Discov.

[25] Zhang S (2016). The potent ALK inhibitor Brigatinib (AP26113) overcomes mechanisms of resistance to First- and second-generation ALK inhibitors in preclinical models. Clin Cancer Res.

[26] Hallberg B, Palmer RH (2013). Mechanistic insight into ALK receptor tyrosine kinase in human cancer biology. Nat Rev Cancer.

[27] Li Y et al (2021) *Endogenous biomarkers for SLC transporter-mediated drug-drug Interaction evaluation*.Molecules, 26(18) 10.3390/molecules26185500 PMC8466752 34576971

[28] Liu X (2019). Overview: role of Drug Transporters in Drug Disposition and its clinical significance. Adv Exp Med Biol.

[29] Giacomini KM (2010). Membrane transporters in drug development. Nat Rev Drug Discov.

[30] Liang Y, Li S, Chen L (2015). The physiological role of drug transporters. Protein Cell.

[31] administration TU (2020) S.F.d., *In Vitro Drug Interaction Studies-Cytochrome P450 Enzyme- and Transporter-Mediated Drug Interactions Guidance for Industry*.

[32] agency Em (2012) *Guideline on the investigation of drug interactions*.

[33] Garrison DA et al (2020) *Role of OATP1B1 and OATP1B3 in drug-drug interactions mediated by tyrosine kinase inhibitors*.Pharmaceutics, 12(9) 10.3390/pharmaceutics12090856 PMC7559291 32916864

[34] Shi Y (2022). Safety and activity of WX-0593 (Iruplinalkib) in patients with ALK- or ROS1-rearranged advanced non-small cell lung cancer: a phase 1 dose-escalation and dose-expansion trial. Signal Transduct Target Ther.

[35] Zhang J et al (2016) *ROS and ROS-Mediated Cellular Signaling* Oxid Med Cell Longev, 2016: p. 4350965 10.1155/2016/4350965 PMC4779832 26998193

[36] Mok T (2020). Updated overall survival and final progression-free survival data for patients with treatment-naive advanced ALK-positive non-small-cell lung cancer in the ALEX study. Ann Oncol.

[37] Camidge DR (2020). Brigatinib Versus Crizotinib in Advanced ALK inhibitor-naive ALK-Positive Non-Small Cell Lung Cancer: second interim analysis of the phase III ALTA-1L trial. J Clin Oncol.

[38] *NCCN Clinical Practice Guidelines in Oncology (NCCN Guidelines). Non-Small Cell Lung Cancer. Version 1.2022*. Fort Washington:National Comprehensive Cancer Network

[39] Gainor JF (2016). Molecular Mechanisms of Resistance to First- and second-generation ALK inhibitors in ALK-Rearranged Lung Cancer. Cancer Discov.

[40] Shaw AT (2019). ALK Resistance mutations and efficacy of Lorlatinib in Advanced anaplastic lymphoma kinase-positive non-small-cell Lung Cancer. J Clin Oncol.

[41] Gainor JF et al (2017) *Patterns of Metastatic Spread and Mechanisms of Resistance to Crizotinib in ROS1-Positive Non-Small-Cell Lung Cancer* JCO Precis Oncol, 2017 10.1200/PO.17.00063 PMC5766287 29333528

[42] Keddy C (2022). Resistance Profile and Structural modeling of Next-Generation ROS1 tyrosine kinase inhibitors. Mol Cancer Ther.

[43] Lin JJ (2021). Spectrum of mechanisms of resistance to Crizotinib and Lorlatinib in ROS1 Fusion-Positive Lung Cancer. Clin Cancer Res.

[44] Liu X (2022). Discovery and preclinical evaluations of WX-0593, a novel ALK inhibitor targeting crizotinib-resistant mutations. Bioorg Med Chem Lett.

[45] Sun Y (2013). ALK inhibitor PF02341066 (crizotinib) increases sensitivity to radiation in non-small cell lung cancer expressing EML4-ALK. Mol Cancer Ther.

[46] Guo Y (2019). Recent progress in Rare Oncogenic Drivers and targeted Therapy for Non-Small Cell Lung Cancer. Onco Targets Ther.

[47] Drilon A (2021). ROS1-dependent cancers - biology, diagnostics and therapeutics. Nat Rev Clin Oncol.

[48] Chiarle R (2008). The anaplastic lymphoma kinase in the pathogenesis of cancer. Nat Rev Cancer.

[49] Karar J, Maity A (2011). PI3K/AKT/mTOR pathway in Angiogenesis. Front Mol Neurosci.

[50] Yan D (2013). Polyunsaturated fatty acids promote the expansion of myeloid-derived suppressor cells by activating the JAK/STAT3 pathway. Eur J Immunol.

[51] Zhang X (2019). Hypoxic BMSC-derived exosomal miRNAs promote metastasis of lung cancer cells via STAT3-induced EMT. Mol Cancer.

