# Supplementary material for: Iruplinalkib (WX‑0593), a novel ALK/ROS1 inhibitor, overcomes crizotinib resistance in preclinical models for non-small cell lung cancer
Source: Invest New Drugs. 2023 Apr 10;41(2):254–66. doi: 10.1007/s10637-023-01350-x (PMC10140010; doi:10.1007/s10637-023-01350-x)
Supplement: Supplementary file 1 — Supplementary Material 1 [file 10637_2023_1350_MOESM1_ESM.docx]

**Supplementary Table 1. The sources of cell lines and media used**

| **Cell lines** | **Sources** | **Media** | **Sources** |
| --- | --- | --- | --- |
| Karpas 299 cells | Sigma-Aldrich | RPMI-1640 medium | Gibco |
| NCI-H1975 cells | ATCC | RPMI-1640 medium | Gibco |
| NCI-H3122 cells | Nanjing Kebai Biotechnology Co., Ltd. | RPMI-1640 medium | Gibco |
| Ba/F3 cells | RIKEN BioResource Center | RPMI-1640 medium | Gibco |
| HCC-78 cells | Shanghai Hongshun Biotechnology Co., Ltd. | IMDM medium | Gibco |
| NIH-3T3 (CD74-ROS1) cells | Nanjing GenScript Biotechnology Co., Ltd. | DMEM medium | Gibco |
| Ba/F3 (SLC34A2-ROS1) cells | Kyinno Biotechnology (Beijing) Co., Ltd. | RPMI-1640 medium | Gibco |
| Caco-2 cells | ATCC | MEM | Gibco |
| HEK293 cells | Genomembrane | DMEM-HG | Gibco |

Note: RPMI: Roswell Park Memorial Institute; IMDM: Iscove's modified Dulbecco's medium; DMEM: Dulbecco's modified eagle medium; MEM, Minimum Essential Medium; DMEM-HG: Dulbecco's modified eagle medium high glucose.

**Supplementary Table 2. Antibodies and sources used in western blot**

| **Antibody (dilution)** | **Company** |
| --- | --- |
| anti-GAPDH mouser McAb (1:50000-1:500000) | Proteintech |
| Anti-ROS1 (phospho Y2114) (1:1000) | Abcam |
| ROS1 (D4D6®) Rabbit mAb (1:1000) | Cell Signaling Technology |
| ALK(D5F3) XP(R) Rabbit mAb (1:2000) | Cell Signaling Technology |
| p-ALK(Y1604) Rabbit mAb (1:1000) | Cell Signaling Technology |
| Stat3(79D7) Rabbit mAb (1:2000) | Cell Signaling Technology |
| P-Stat3(Y705)(D3A7) XP(R)Rabbit mAb (1:2000) | Cell Signaling Technology |
| P-Stat 5(Y694) (C11C5) Rabbit mAb (1:1000) | Cell Signaling Technology |
| p44/42 MAPK (Erk1/2) Rabbit Ab (1:1000) | Cell Signaling Technology |
| P-p44/42 MAPK (T202/Y204)(197G2) Rabbit mAb (1:2000) | Cell Signaling Technology |
| Akt (pan)(C67E7) Rabbit mAb (1:1000) | Cell Signaling Technology |
| P-Akt (S473)(D9E) XP(R) Rabbit mAb (1:2000) | Cell Signaling Technology |
| Anti-rabbit IgG, HRP-linked Antibody (1:1000-1:3000) | Cell Signaling Technology |
| Anti-mouse IgG, HRP-linked Antibody (1:1000-1:3000) | Cell Signaling Technology |

Note: Ab: antibody; ALK: anaplastic lymphoma kinase; GAPDH: glyceraldehyde-3-phosphate dehydrogenase; HRP: horseradish peroxidase; IgG: immunoglobulin G; MAPK: mitogen-activated protein kinase; ROS1: *ROS* proto-oncogene 1, receptor tyrosine kinase; SHP: src-homology domain 2 (SH2) -containing protein tyrosine phosphatase.

**Supplementary Table 3. IC_50_ values for the inhibitory effects of iruplinalkib and brigatinib on 27 kinases**

| **Targets** | **IC_50_ (nM)** | |
| --- | --- | --- |
|  | **Iruplinalkib** | **Brigatinib** |
| ABL | 576 ± 108 | 596 ± 52 |
| AUR2 | 1085 ± 106 | 119 ± 13 |
| CaMK2 alpha | 939 ± 99 | 319 ± 13 |
| CDK2 | 1564 ± 1125 | 1511 ± 554 |
| CHK1 | 185 ± 41 | 70 ± 32 |
| CK1 alpha | > 10000 | > 10000 |
| c-MET | 2937 ± 232 | 1827 ± 231 |
| DYRK3 | 8014 ± 583 | > 10000 |
| EGFR wild type | 35 ± 10 | 31 ± 2 |
| EPHA2 | > 10000 | > 10000 |
| FGFR1 | 249 ± 71 | 109 ± 33 |
| GSK3 beta | > 10000 | > 10000 |
| INSR | 699 ± 261 | 626 ± 304 |
| JAK2 | 595 ± 12 | 680 ± 47 |
| JNK1 | 2038 ± 1166 | 1520 ± 733 |
| KDR | 260 ± 128 | 178 ± 52 |
| LCK | 825 ± 243 | 444 ± 84 |
| MAP4K4 | 1841 ± 679 | 1681 ± 762 |
| MAPKAPK2 | > 10000 | > 10000 |
| MINK | 3893 ± 1060 | 4037 ± 2157 |
| MST4 | 1398 ± 251 | 1083 ± 502 |
| P38 alpha | > 10000 | > 10000 |
| PDK1 | > 10000 | > 10000 |
| PKA | > 10000 | > 10000 |
| SRC | 1683 ± 872 | 950 ± 309 |
| TAOK2 | 1503 ± 442 | 964 ± 48 |
| TNIK | 3208 ± 1541 | 2371 ± 1328 |

Note: IC_50_, half maximal inhibitory concentration; ABL: Abelson tyrosine kinase; AUR2: Aurora kinase 2; CaMK2: Calcium/Calmodulin Dependent Protein Kinase II; CDK2: Cyclin Dependent Kinase 2; CHK1: Checkpoint Kinase 1; CK1: Casein Kinase 1; c-MET: c-Mesenchymal-epithelial transition factor; DYRK3: Dual Specificity Tyrosine-(Y)-Phosphorylation Regulated Kinase 3; EGFR: Epidermal Growth Factor Receptor; EPHA2: EPH receptor A2; FGFR1: Fibroblast Growth Factor Receptor 1; GSK3: Glycogen Synthase Kinase 3; INSR: Insulin Receptor; JAK2: Janus Kinase 2; JNK1: c-Jun N-terminal Kinase 1; KDR: Kinase Insert Domain Receptor; LCK: Lymphocyte Cell-Specific Protein-Tyrosine Kinase; MAP4K4: Mitogen-Activated Protein Kinase Kinase Kinase Kinase 4; MAPKAPK2: Mitogen-Activated Protein Kinase-Activated Protein Kinase 2; MINK: Misshapen Like Kinase; MST4: Serine/Threonine Kinase 26; PDK1: Pyruvate Dehydrogenase Kinase 1; PKA: protein kinase A; SRC: SRC Proto-Oncogene, Non-Receptor Tyrosine Kinase; TAOK2: Thousand And One Amino Acid Protein Kinase 2; TNIK: TRAF2 And NCK Interacting Kinase.

**Supplementary Table 4. Tumor volume of iruplinalkib in a crizotinib resistant LU-01-0015 (*HIP-ALK*) patient derived lung-tumor xenografts in nude mice**

| **Group** | **No.** | **Days after administration** | | | | | | | | |
| --- | --- | --- | --- | --- | --- | --- | --- | --- | --- | --- |
|  |  | **0** | **3** | **7** | **10** | **14** | **17** | **21** | **24** | **27** |
| Group 1  Blank | 618 | 165 | 193 | 325 | 364 | 455 | 477 | 774 | 917 | 941 |
|  | 670 | 121 | 127 | 138 | 159 | 169 | 180 | 268 | 281 | 302 |
|  | 672 | 86 | 96 | 118 | 121 | 133 | 150 | 220 | 224 | 247 |
|  | 675 | 109 | 114 | 123 | 142 | 147 | 229 | 352 | 382 | 540 |
|  | 689 | 124 | 159 | 232 | 244 | 384 | 663 | 852 | 1111 | 1253 |
|  | 695 | 231 | 244 | 433 | 682 | 688 | 935 | 1514 | 1734 | 2111 |
|  | 699 | 192 | 227 | 257 | 349 | 432 | 540 | 919 | 603 | 352 |
|  | 730 | 153 | 166 | 274 | 313 | 530 | 617 | 965 | 1111 | 1176 |
|  | 733 | 182 | 247 | 360 | 388 | 423 | 492 | 802 | 841 | 1099 |
|  | Mean | 151 | 175 | 251 | 307 | 374 | 476 | 741 | 801 | 891 |
|  | SEM | 15 | 19 | 37 | 58 | 63 | 85 | 137 | 162 | 201 |
| Group 2  Vehicle  PO  QD×4 W | 604 | 114 | 124 | 216 | 232 | 265 | 383 | 550 | 578 | 711 |
|  | 608 | 159 | 171 | 227 | 297 | 339 | 445 | 548 | 624 | 680 |
|  | 629 | 201 | 285 | 293 | 354 | 498 | 830 | 943 | 1137 | 1465 |
|  | 640 | 221 | 237 | 248 | 316 | 324 | 485 | 840 | 693 | 866 |
|  | 644 | 96 | 138 | 255 | 408 | 516 | 728 | 1199 | 1366 | 1520 |
|  | 650 | 190 | 240 | 328 | 341 | 444 | 525 | 744 | 752 | 810 |
|  | 669 | 144 | 195 | 256 | 299 | 375 | 584 | 725 | 780 | 830 |
|  | 688 | 100 | 129 | 202 | 217 | 248 | 297 | 497 | 527 | 758 |
|  | 732 | 131 | 135 | 140 | 231 | 269 | 280 | 319 | 329 | 342 |
|  | Mean | 150 | 184 | 241 | 300 | 364 | 506 | 707 | 754 | 887 |
|  | SEM | 15 | 20 | 18 | 21 | 34 | 62 | 88 | 106 | 125 |
| Group 3  Brigatinib  10 mg/kg  PO  QD×4 W | 635 | 229 | 206 | 195 | 236 | 204 | 239 | 247 | 262 | 332 |
|  | 638 | 201 | 208 | 180 | 171 | 172 | 176 | 182 | 186 | 197 |
|  | 648 | 134 | 136 | 121 | 101 | 98 | 118 | 156 | 98 | 131 |
|  | 673 | 173 | 143 | 110 | 97 | 118 | 196 | 187 | 210 | 249 |
|  | 691 | 152 | 125 | 74 | 63 | 60 | 66 | 61 | 62 | 47 |
|  | 705 | 164 | 137 | 110 | 85 | 84 | 106 | 96 | 101 | 95 |
|  | 717 | 80 | 82 | 78 | 89 | 74 | 106 | 111 | 137 | 163 |
|  | 744 | 99 | 123 | 152 | 106 | 89 | 97 | 68 | 102 | 85 |
|  | 753 | 111 | 106 | 89 | 80 | 71 | 89 | 85 | 70 | 70 |
|  | Mean | 149 | 141 | 123 | 114 | 108 | 133 | 133 | 136 | 152 |
|  | SEM | 16 | 14 | 15 | 18 | 16 | 19 | 21 | 23 | 31 |
| Group 4  Brigatinib  5 mg/kg  PO  QD×4 W | 622 | 189 | 172 | 149 | 128 | 154 | 177 | 138 | 122 | 113 |
|  | 627 | 117 | 123 | 98 | 67 | 64 | 96 | 255 | 117 | 101 |
|  | 647 | 124 | 114 | 81 | 87 | 86 | 88 | 63 | 57 | 56 |
|  | 651 | 208 | 201 | 209 | 178 | 153 | 240 | 191 | 227 | 235 |
|  | 696 | 95 | 102 | 79 | 76 | 70 | 88 | 91 | 67 | 69 |
|  | 707 | 164 | 185 | 170 | 131 | 122 | 136 | 187 | 172 | 214 |
|  | 710 | 100 | 96 | 83 | 72 | 70 | 80 | 63 | 63 | 69 |
|  | 739 | 154 | 160 | 126 | 97 | 85 | 99 | 100 | 91 | 105 |
|  | 758 | 221 | 275 | 321 | 265 | 299 | 310 | 368 | 412 | 446 |
|  | Mean | 153 | 159 | 146 | 122 | 123 | 146 | 162 | 148 | 157 |
|  | SEM | 16 | 19 | 26 | 21 | 25 | 27 | 34 | 38 | 42 |
| Group 5  Crizotinib  20 mg/kg  PO  QD×4 W | 611 | 127 | 150 | 180 | 135 | 170 | 195 | 149 | 128 | 135 |
|  | 615 | 105 | 114 | 143 | 141 | 167 | 135 | 188 | 141 | 146 |
|  | 628 | 204 | 225 | 170 | 152 | 169 | 182 | 288 | 322 | 401 |
|  | 641 | 144 | 188 | 174 | 345 | 347 | 436 | 567 | 478 | 561 |
|  | 643 | 118 | 125 | 132 | 163 | 179 | 180 | 139 | 151 | 158 |
|  | 653 | 170 | 216 | 223 | 248 | 244 | 248 | 278 | 410 | 484 |
|  | 664 | 82 | 103 | 111 | 122 | 105 | 132 | 129 | 79 | 86 |
|  | 703 | 184 | 213 | 413 | 416 | 400 | 458 | 604 | 608 | 821 |
|  | 731 | 225 | 249 | 283 | 252 | 200 | 233 | 240 | 253 | 316 |
|  | Mean | 151 | 176 | 203 | 219 | 220 | 244 | 287 | 285 | 345 |
|  | SEM | 16 | 18 | 31 | 35 | 32 | 40 | 60 | 61 | 82 |
| Group 6  Crizotinib  10 mg/kg  PO  QD×4 W | 620 | 107 | 138 | 138 | 172 | 208 | 214 | 265 | 254 | 269 |
|  | 623 | 202 | 231 | 345 | 424 | 419 | 447 | 677 | 862 | 1288 |
|  | 632 | 241 | 262 | 348 | 446 | 629 | 824 | 1155 | 1327 | 1557 |
|  | 636 | 151 | 210 | 360 | 320 | 408 | 542 | 747 | 1120 | 1211 |
|  | 637 | 79 | 122 | 181 | 206 | 312 | 456 | 642 | 433 | 540 |
|  | 697 | 168 | 219 | 237 | 345 | 463 | 585 | 820 | 867 | 996 |
|  | 718 | 122 | 134 | 168 | 288 | 321 | 380 | 699 | 696 | 814 |
|  | 723 | 170 | 173 | 280 | 319 | 535 | 597 | 1069 | 990 | 1065 |
|  | 750 | 127 | 138 | 139 | 163 | 243 | 319 | 578 | 358 | 283 |
|  | Mean | 152 | 181 | 244 | 298 | 393 | 485 | 739 | 767 | 891 |
|  | SEM | 17 | 17 | 31 | 34 | 46 | 60 | 88 | 121 | 150 |
| Group 7  Iruplinalkib  15 mg/kg  PO  QD×4 W | 602 | 101 | 81 | 51 | 48 | 41 | 44 | 35 | 29 | 30 |
|  | 624 | 162 | 117 | 61 | 61 | 57 | 92 | 87 | 56 | 67 |
|  | 634 | 129 | 84 | 57 | 44 | 43 | 41 | 45 | 37 | 38 |
|  | 660 | 99 | 63 | 56 | 35 | 31 | 24 | 30 | 23 | 27 |
|  | 680 | 217 | 150 | 78 | 63 | 72 | 68 | 53 | 45 | 62 |
|  | 709 | 147 | 118 | 72 | 79 | 46 | 47 | 59 | 53 | 54 |
|  | 729 | 109 | 79 | 52 | 48 | 43 | 32 | 37 | 34 | 44 |
|  | 745 | 207 | 154 | 71 | 45 | 44 | 42 | 41 | 22 | 42 |
|  | 760 | 174 | 190 | 98 | 63 | 60 | 68 | 57 | 91 | 78 |
|  | Mean | 149 | 115 | 66 | 54 | 49 | 51 | 49 | 43 | 49 |
|  | SEM | 15 | 14 | 5 | 5 | 4 | 7 | 6 | 7 | 6 |
| Group 8  Iruplinalkib  10 mg/kg (D0-D7)/2.5 mg/kg  PO  QD×4 W | 607 | 136 | 115 | 71 | 49 | 48 | 58 | 56 | 57 | 65 |
|  | 614 | 117 | 76 | 51 | 43 | 61 | 56 | 80 | 98 | 99 |
|  | 631 | 124 | 84 | 65 | 67 | 69 | 74 | 107 | 101 | 128 |
|  | 646 | 206 | 182 | 97 | 45 | 40 | 57 | 68 | 58 | 75 |
|  | 674 | 108 | 81 | 62 | 38 | 38 | 46 | 64 | 47 | 62 |
|  | 693 | 166 | 136 | 77 | 74 | 82 | 131 | 139 | 170 | 165 |
|  | 734 | 187 | 148 | 81 | 60 | 58 | 71 | 135 | 88 | 112 |
|  | 737 | 216 | 189 | 114 | 133 | 138 | 157 | 257 | 287 | 372 |
|  | 746 | 94 | 110 | 74 | 32 | 30 | 45 | 55 | 51 | 68 |
|  | Mean | 150 | 124 | 77 | 60 | 63 | 77 | 107 | 106 | 127 |
|  | SEM | 15 | 14 | 6 | 10 | 11 | 13 | 22 | 26 | 33 |
| Group 9  Iruplinalkib  5 mg/kg  PO  QD×4 W | 601 | 104 | 92 | 60 | 74 | 65 | 78 | 103 | 112 | 128 |
|  | 642 | 186 | 162 | 101 | 114 | 98 | 123 | 122 | 117 | 131 |
|  | 663 | 123 | 92 | 50 | 72 | 56 | 47 | 32 | 30 | 39 |
|  | 665 | 148 | 110 | 81 | 73 | 66 | 64 | 67 | 87 | 109 |
|  | 678 | 195 | 166 | 85 | 72 | 68 | 102 | 98 | 79 | 93 |
|  | 684 | 89 | 75 | 53 | 66 | 58 | 59 | 58 | 45 | 46 |
|  | 694 | 217 | 190 | 143 | 103 | 94 | 83 | 107 | 94 | 95 |
|  | 735 | 117 | 91 | 65 | 55 | 49 | 81 | 90 | 84 | 99 |
|  | 736 | 165 | 165 | 96 | 80 | 75 | 96 | 121 | 118 | 104 |
|  | Mean | 149 | 127 | 82 | 79 | 70 | 81 | 89 | 85 | 94 |
|  | SEM | 15 | 14 | 10 | 6 | 6 | 8 | 10 | 10 | 11 |

Note: SEM: standard error of mean.

**Supplementary Table 5. Tumor volume of NCI-H3122** **(*EML4-ALK*) xenograft model in nude mice.**

| **Group** | **No.** | **Days after administration** | | | | | | |
| --- | --- | --- | --- | --- | --- | --- | --- | --- |
|  |  | **0** | **3** | **6** | **9** | **13** | **16** | **20** |
| Group 1  Blank | 101 | 187 | 404 | 568 | 886 | 1153 | 1453 | 1937 |
|  | 102 | 159 | 352 | 534 | 785 | 1142 | 1378 | 1733 |
|  | 103 | 142 | 305 | 405 | 641 | 865 | 896.6 | 1233 |
|  | 104 | 248 | 479 | 731 | 925 | 1419 | 1579 | 1983 |
|  | 105 | 167 | 393 | 599 | 1087 | 1265 | 1612 | 2080 |
|  | 106 | 195 | 294 | 395 | 544 | 626 | 793 | 1180 |
|  | 107 | 165 | 309 | 516 | 737 | 951 | 1114 | 1445 |
|  | 108 | 160 | 302 | 513 | 699 | 863 | 1039 | 1439 |
|  | 109 | 146 | 219 | 363 | 601 | 832 | 1102 | 1360 |
|  | Mean | 174 | 340 | 514 | 767 | 1013 | 1219 | 1599 |
|  | SD | 33 | 76 | 115 | 174 | 249 | 297 | 340 |
| Group 2  Brigatinib  5mg/kg  PO  QD×21 | 201 | 159 | 235 | 344 | 473 | 454 | 521 | 710 |
|  | 202 | 116 | 195 | 246 | 337 | 241 | 320 | 414 |
|  | 203 | 221 | 320 | 444 | 548 | 654 | 707 | 729 |
|  | 204 | 152 | 239 | 301 | 418 | 480 | 541 | 637 |
|  | 205 | 134 | 193 | 248 | 289 | 315 | 350 | 412 |
|  | 206 | 154 | 260 | 364 | 518 | 575 | 627 | 711 |
|  | 207 | 194 | 247 | 372 | 482 | 637 | 656 | 707 |
|  | 208 | 207 | 262 | 370 | 535 | 495 | 464 | 501 |
|  | 209 | 229 | 319 | 474 | 555 | 666 | 737 | 687 |
|  | Mean | 174 | 252 | 352 | 461 | 502 | 547 | 612 |
|  | SD | 40 | 45 | 78 | 95 | 150 | 149 | 132 |
| Group 3  Iruplinalkib  2.5mg/kg  PO  QD×21 | 301 | 188 | 324 | 431 | 596 | 630 | 729 | 760 |
|  | 302 | 164 | 257 | 392 | 541 | 635 | 821 | 976 |
|  | 303 | 230 | 368 | 594 | 738 | 849 | 1141 | 1087 |
|  | 304 | 150 | 284 | 476 | 570 | 718 | 926 | 1021 |
|  | 305 | 236 | 366 | 695 | 1128 | 1127 | 1167 | 1125 |
|  | 306 | 136 | 229 | 327 | 398 | 586 | 805 | 811 |
|  | 307 | 182 | 324 | 410 | 498 | 573 | 883 | 1040 |
|  | 308 | 147 | 267 | 337 | 499 | 580 | 751 | 1032 |
|  | 309 | 147 | 234 | 384 | 486 | 545 | 758.9 | 895 |
|  | Mean | 176 | 295 | 449 | 606 | 694 | 887 | 972 |
|  | SD | 37 | 53 | 122 | 217 | 188 | 164 | 125 |
| Group 4  Iruplinalkib  5mg/kg  PO  QD×21 | 401 | 233 | 306 | 537 | 567 | 666 | 718 | 811 |
|  | 402 | 239 | 299 | 487 | 541 | 699 | 880 | 875 |
|  | 403 | 173 | 256 | 339 | 407 | 523 | 646 | 702 |
|  | 404 | 121 | 223 | 249 | 332 | 388 | 506 | 717 |
|  | 405 | 155 | 292 | 357 | 403 | 515 | 603 | 714 |
|  | 406 | 235 | 296 | 438 | 534 | 655 | 661 | 785 |
|  | 407 | 148 | 213 | 342 | 389 | 478 | 560 | 652 |
|  | 408 | 158 | 211 | 328 | 344 | 427 | 536 | 643 |
|  | 409 | 130 | 249 | 258 | 356 | 426 | 508 | 639 |
|  | Mean | 177 | 260 | 371 | 430 | 531 | 624 | 727 |
|  | SD | 46 | 39 | 98 | 92 | 116 | 120 | 82 |
| Group 5  Iruplinalkib  10mg/kg  PO  QD×21 | 501 | 173 | 226 | 227 | 279 | 281 | 277 | 345 |
|  | 502 | 162 | 136 | 145 | 162 | 172 | 122 | 115 |
|  | 503 | 223 | 257 | 273 | 355 | 338 | 294 | 298 |
|  | 504 | 169 | 185 | 218 | 226 | 218 | 186 | 197 |
|  | 505 | 163 | 158 | 198 | 259 | 251 | 241 | 270 |
|  | 506 | 236 | 264 | 295 | 326 | 343 | 333 | 324 |
|  | 507 | 168 | 194 | 193 | 241 | 263 | 205 | 196 |
|  | 508 | 140 | 143 | 151 | 150 | 159 | 166 | 200 |
|  | 509 | 130 | 148 | 132 | 160 | 160 | 141 | 150 |
|  | Mean | 174 | 190 | 204 | 240 | 243 | 218 | 233 |
|  | SD | 35 | 49 | 56 | 74 | 71 | 73 | 80 |

Note: SD: standard deviation.

**Supplementary Table 6. Pre-test results of iruplinalkib as OATP1B1 substrate and inhibitor investigation**

| **Cell lines** | **Reference or examination of inhibitors** | | **Criteria or examination of inhibitors** | | **Uptake transport activity (pmol/mg protein/min)** | | | | | | **Ratio of uptake transit activity** | **Inhibition rate (%)** |
| --- | --- | --- | --- | --- | --- | --- | --- | --- | --- | --- | --- | --- |
|  | **Compound** | **Conc (μM)** | **Compound** | **Conc (μM)** | **Repeat 1** | **Repeat 2** | **Mean** | **SD** | **CV (%)** | **Net value** |  |  |
| MOCK293 | — | — | ATV | 1 | 0.186 | 0.140 | 0.163 | 0.023 | 14.1 | 0.359 | 3.20 | — |
| OATP1B1-293 |  |  |  |  | 0.506 | 0.537 | 0.522 | 0.016 | 2.97 |  |  |  |
| MOCK293 | CsA | 10 |  |  | 0.239 | 0.286 | 0.263 | 0.024 | 8.95 | 0.232 | 1.88 | 35.4 |
| OATP1B1-293 |  |  |  |  | 0.532 | 0.456 | 0.494 | 0.038 | 7.69 |  |  |  |
| MOCK293 | — | — | Iruplinalkib | 10 | 100.4 | 103.6 | 102.0 | 1.619 | 1.59 | -46.9 | 0.54 | — |
| OATP1B1-293 |  |  |  |  | 50.74 | 59.41 | 55.08 | 4.338 | 7.88 |  |  |  |
| MOCK293 | Iruplinalkib | 50 | ATV | 1 | 0.099 | 0.109 | 0.104 | 0.005 | 4.81 | 0.116 | 2.11 | 67.8 |
| OATP1B1-293 |  |  |  |  | 0.218 | 0.221 | 0.220 | 0.002 | 0.68 |  |  |  |
| MOCK293 |  | 5 |  |  | 0.086 | 0.130 | 0.108 | 0.022 | 20.4 | 0.255 | 3.36 | 28.9 |
| OATP1B1-293 |  |  |  |  | 0.345 | 0.381 | 0.363 | 0.018 | 4.96 |  |  |  |
| MOCK293 |  | 0.5 |  |  | 0.063 | 0.114 | 0.089 | 0.026 | 28.8 | 0.264 | 3.98 | 26.5 |
| OATP1B1-293 |  |  |  |  | 0.340 | 0.364 | 0.352 | 0.012 | 3.41 |  |  |  |

Note: SD, standard deviation; CV, coefficient of variation.

**Supplementary Table 7. Pre-test results of iruplinalkib as OATP1B3 substrate and inhibitor investigation**

| **Cell lines** | **Reference or examination of inhibitors** | | **Criteria or examination of inhibitors** | | **Uptake transport activity (pmol/mg protein/min)** | | | | | | **Ratio of uptake transit activity** | **Inhibition rate (%)** |
| --- | --- | --- | --- | --- | --- | --- | --- | --- | --- | --- | --- | --- |
|  | **Compound** | **Conc (μM)** | **Compound** | **Conc (μM)** | **Repeat 1** | **Repeat 2** | **Mean** | **SD** | **CV (%)** | **Net value** |  |  |
| MOCK293 | — | — | ATV | 1 | 2.810 | 3.250 | 3.030 | 0.220 | 7.26 | 10.6 | 4.49 | — |
| OATP1B1-293 |  |  |  |  | 14.30 | 12.90 | 13.60 | 0.690 | 5.06 |  |  |  |
| MOCK293 | CsA | 10 |  |  | 8.220 | 7.160 | 7.690 | 0.528 | 6.86 | -0.18 | 0.98 | 101.7 |
| OATP1B1-293 |  |  |  |  | 7.640 | 7.370 | 7.510 | 0.135 | 1.80 |  |  |  |
| MOCK293 | — | — | Iruplinalkib | 10 | 221.3 | 239.2 | 230.3 | 8.971 | 3.90 | 97.7 | 1.42 | — |
| OATP1B1-293 |  |  |  |  | 313.3 | 342.6 | 327.9 | 14.63 | 4.46 |  |  |  |
| MOCK293 | Iruplinalkib | 50 | ATV | 1 | 5.480 | 5.730 | 5.610 | 0.127 | 2.27 | 6.24 | 2.11 | 41.1 |
| OATP1B1-293 |  |  |  |  | 11.50 | 12.20 | 11.90 | 0.380 | 3.21 |  |  |  |
| MOCK293 |  | 5 |  |  | 4.820 | 4.360 | 4.590 | 0.228 | 4.95 | 8.98 | 2.96 | 15.3 |
| OATP1B1-293 |  |  |  |  | 15.30 | 11.80 | 13.60 | 1.744 | 12.8 |  |  |  |
| MOCK293 |  | 0.5 |  |  | 3.030 | 3.030 | 3.030 | 0.002 | 0.08 | 9.84 | 4.25 | 7.2 |
| OATP1B1-293 |  |  |  |  | 12.90 | 12.80 | 12.90 | 0.043 | 0.33 |  |  |  |

Note: SD, standard deviation; CV, coefficient of variation.

**Supplementary Table 8. Pre-test results of iruplinalkib as OAT1 substrate and inhibitor investigation**

| **Cell lines** | **Reference or examination of inhibitors** | | **Criteria or examination of inhibitors** | | **Uptake transport activity (pmol/mg protein/min)** | | | | | | **Ratio of uptake transit activity** | **Inhibition rate (%)** |
| --- | --- | --- | --- | --- | --- | --- | --- | --- | --- | --- | --- | --- |
|  | **Compound** | **Conc (μM)** | **Compound** | **Conc (μM)** | **Repeat 1** | **Repeat 2** | **Mean** | **SD** | **CV (%)** | **Net value** |  |  |
| MOCK293 | — | — | ATV | 1 | 3.281 | 3.505 | 3.393 | 0.112 | 3.30 | 28.01 | 9.26 | — |
| OATP1B1-293 |  |  |  |  | 36.42 | 26.39 | 31.40 | 5.014 | 16.0 |  |  |  |
| MOCK293 | CsA | 10 |  |  | 3.331 | 3.434 | 3.382 | 0.051 | 1.52 | 14.03 | 5.15 | 49.9 |
| OATP1B1-293 |  |  |  |  | 17.62 | 17.21 | 17.42 | 0.202 | 1.16 |  |  |  |
| MOCK293 | — | — | Iruplinalkib | 10 | 302.7 | 324.5 | 313.6 | 10.88 | 3.47 | -24.51 | 0.92 | — |
| OATP1B1-293 |  |  |  |  | 285.7 | 292.5 | 289.1 | 3.395 | 1.17 |  |  |  |
| MOCK293 | Iruplinalkib | 50 | ATV | 1 | 7.193 | 5.433 | 6.313 | 0.880 | 13.9 | 19.07 | 4.02 | 31.9 |
| OATP1B1-293 |  |  |  |  | 25.80 | 24.96 | 25.38 | 0.418 | 1.65 |  |  |  |
| MOCK293 |  | 5 |  |  | 7.190 | 5.430 | 6.310 | 0.880 | 13.9 | 21.70 | 4.44 | 22.5 |
| OATP1B1-293 |  |  |  |  | 27.56 | 28.45 | 28.01 | 0.444 | 1.59 |  |  |  |
| MOCK293 |  | 0.5 |  |  | 6.224 | 5.604 | 5.914 | 0.310 | 5.25 | 20.52 | 4.47 | 26.7 |
| OATP1B1-293 |  |  |  |  | 26.08 | 26.79 | 26.43 | 0.359 | 1.36 |  |  |  |

Note: SD, standard deviation; CV, coefficient of variation.

**Supplementary Table 9. Pre-test results of iruplinalkib as OAT3 substrate and inhibitor investigation**

| **Cell lines** | **Reference or examination of inhibitors** | | **Criteria or examination of inhibitors** | | **Uptake transport activity (pmol/mg protein/min)** | | | | | | **Ratio of uptake transit activity** | **Inhibition rate (%)** |
| --- | --- | --- | --- | --- | --- | --- | --- | --- | --- | --- | --- | --- |
|  | **Compound** | **Conc (μM)** | **Compound** | **Conc (μM)** | **Repeat 1** | **Repeat 2** | **Mean** | **SD** | **CV (%)** | **Net value** |  |  |
| MOCK293 | — | — | ATV | 1 | 59.57 | 90.74 | 75.16 | 15.58 | 20.7 | 652.7 | 9.26 | — |
| OATP1B1-293 |  |  |  |  | 731.6 | 724.2 | 727.9 | 3.681 | 0.51 |  |  |  |
| MOCK293 | CsA | 10 |  |  | 373.3 | 144.8 | 259.1 | 114.3 | 44.1 | 195.2 | 5.15 | 49.9 |
| OATP1B1-293 |  |  |  |  | 472.5 | 436.0 | 454.3 | 18.22 | 4.01 |  |  |  |
| MOCK293 | — | — | Iruplinalkib | 10 | 309.8 | 378.3 | 344.0 | 34.24 | 9.95 | -255.5 | 0.92 | — |
| OATP1B1-293 |  |  |  |  | 81.45 | 95.69 | 88.57 | 7.120 | 8.04 |  |  |  |
| MOCK293 | Iruplinalkib | 50 | ATV | 1 | 162.6 | 146.1 | 154.3 | 8.202 | 5.31 | 682.6 | 4.02 | 31.9 |
| OATP1B1-293 |  |  |  |  | 878.3 | 795.6 | 836.9 | 41.37 | 4.94 |  |  |  |
| MOCK293 |  | 5 |  |  | 145.2 | 137.3 | 141.2 | 3.980 | 2.82 | 700.4 | 4.44 | 22.5 |
| OATP1B1-293 |  |  |  |  | 848.0 | 835.2 | 841.6 | 6.400 | 0.76 |  |  |  |
| MOCK293 |  | 0.5 |  |  | 223.0 | 141.0 | 182.0 | 40.98 | 22.5 | 626.9 | 4.47 | 26.7 |
| OATP1B1-293 |  |  |  |  | 792.0 | 825.8 | 808.9 | 16.93 | 2.09 |  |  |  |

Note: SD, standard deviation; CV, coefficient of variation.

**Supplementary Table 10. Pre-test results of iruplinalkib as MATE1 substrate and inhibitor investigation**

| **Cell lines** | **Reference or examination of inhibitors** | | **Criteria or examination of inhibitors** | | **Uptake transport activity (pmol/mg protein/min)** | | | | | | **Ratio of uptake transit activity** | **Inhibition rate (%)** |
| --- | --- | --- | --- | --- | --- | --- | --- | --- | --- | --- | --- | --- |
|  | **Compound** | **Conc (μM)** | **Compound** | **Conc (μM)** | **Repeat 1** | **Repeat 2** | **Mean** | **SD** | **CV (%)** | **Net value** |  |  |
| MOCK293 | — | — | MPP | 10 | 163.5 | 175.3 | 169.4 | 5.945 | 3.51 | 993.3 | 6.86 |  |
| MATE1-293 |  |  |  |  | 942.2 | 1383.2 | 1162.7 | 220.5 | 19.0 |  |  |  |
| MOCK293 | Pyrimethamine | 2 |  |  | 358.7 | 287.2 | 322.9 | 35.77 | 11.1 | -226.6 | 0.30 | 122.8 |
| MATE1-293 |  |  |  |  | 103.1 | 89.47 | 96.31 | 6.835 | 7.10 |  |  |  |
| MOCK293 | Iruplinalkib | 50 |  |  | 374.2 | 549.4 | 461.8 | 87.58 | 19.0 | -114.5 | 0.75 | 111.5 |
| MATE1-293 |  |  |  |  | 337.1 | 357.5 | 347.3 | 10.22 | 2.94 |  |  |  |
| MOCK293 |  | 5 |  |  | 125.0 | 77.2 | 101.1 | 23.95 | 23.7 | 55.01 | 1.54 | 94.5 |
| MATE1-293 |  |  |  |  | 156.5 | 155.7 | 156.1 | 0.411 | 0.26 |  |  |  |
| MOCK293 |  | 0.5 |  |  | 320.6 | 158.1 | 239.4 | 81.22 | 33.9 | 532.9 | 3.23 | 46.4 |
| MATE1-293 |  |  |  |  | 830.6 | 713.8 | 772.2 | 58.42 | 7.57 |  |  |  |

Note: SD, standard deviation; CV, coefficient of variation.

**Supplementary Table 11. The results of uptake and transport activity of OATP1B1 in different concentrations of iruplinalkib**

| **Cell lines** | **Reference or examination inhibitors** | | **Uptake transport activity (pmol/mg protein/min)** | | | | **Ratio of uptake transit activity** |
| --- | --- | --- | --- | --- | --- | --- | --- |
|  | **Compound** | **Conc (μM)** | **Mean** | **SD** | **CV (%)** | **Net value** |  |
| MOCK293 | ATV | 1 | 4.550 | 0.742 | 16.3 | 12.87 | 3.83 |
| OATP1B1-293 |  |  | 17.42 | 0.472 | 2.71 |  |  |
| MOCK293 | Iruplinalkib | 0.3 | 7.468 | 0.897 | 12.0 | 0.759 | 1.10 |
| OATP1B1-293 |  |  | 8.227 | 1.434 | 17.4 |  |  |
| MOCK293 |  | 3 | 54.18 | 3.532 | 6.52 | -7.440 | 0.86 |
| OATP1B1-293 |  |  | 46.74 | 1.271 | 2.72 |  |  |
| MOCK293 |  | 30 | 601.2 | 32.40 | 5.39 | -36.47 | 0.94 |
| OATP1B1-293 |  |  | 564.7 | 29.70 | 5.26 |  |  |
| MOCK293 | ATV | 1 | 2.359 | 0.244 | 10.3 | 14.00 | 6.93 |
| OATP1B3-293 |  |  | 16.36 | 0.298 | 1.82 |  |  |
| MOCK293 | Iruplinalkib | 0.3 | 5.902 | 1.286 | 21.8 | 2.263 | 1.38 |
| OATP1B3-293 |  |  | 8.165 | 2.341 | 28.7 |  |  |
| MOCK293 |  | 3 | 44.98 | 2.108 | 4.69 | 12.964 | 1.29 |
| OATP1B3-293 |  |  | 57.94 | 2.664 | 4.60 |  |  |
| MOCK293 |  | 30 | 484.5 | 26.02 | 5.37 | 156.58 | 1.32 |
| OATP1B3-293 |  |  | 641.1 | 22.62 | 3.53 |  |  |
| MOCK293 | Metformin | 20 | 3.503 | 0.357 | 10.2 | 79.21 | 23.61 |
| OCT2-293 |  |  | 82.72 | 4.758 | 5.75 |  |  |
| MOCK293 | Iruplinalkib | 0.3 | 7.280 | 0.658 | 9.04 | 0.90 | 1.12 |
| OCT2-293 |  |  | 8.178 | 0.678 | 8.29 |  |  |
| MOCK293 |  | 3 | 80.35 | 2.042 | 2.54 | 7.88 | 1.10 |
| OCT2-293 |  |  | 88.23 | 4.090 | 4.64 |  |  |
| MOCK293 |  | 30 | 999.8 | 24.30 | 2.43 | 166.05 | 1.17 |
| OCT2-293 |  |  | 1165.9 | 43.74 | 3.75 |  |  |

Note: SD, standard deviation; CV, coefficient of variation.

**Supplementary Table 12. The results of iruplinalkib as a BSEP inhibitor**

| **Membrane capsule and treatment** | **Standard substrate** | | **Reference or examination inhibitors** | | **Substrate transfer volume (pmol)** | | | | | **Uptake transport activity (pmol/mg protein/min)** | **Volume of substrate solution transferred (μL/mg protein/min)** | **Inhibition rate (%)** |
| --- | --- | --- | --- | --- | --- | --- | --- | --- | --- | --- | --- | --- |
|  | **Compound** | **Conc (μM)** | **Compound** | **Conc (μM)** | **Mean** | **SD** | **CV (%)** | **Net value** | **ATP-dependent** |  |  |  |
| NC+AMP | TCA | 10 |  |  | 0.255 | 0.090 | 35.4 |  |  |  |  |  |
| NC+ATP |  |  |  |  | 0.150 | 0.039 | 25.9 |  |  |  |  |  |
| BSEP+AMP |  |  |  |  | 0.287 | 0.064 | 22.3 | 0.032 | 4.683 | 18.7 | 1.87 |  |
| BSEP+ATP |  |  |  |  | 4.865 | 0.959 | 19.7 | 4.715 |  |  |  |  |
| BSEP+AMP |  |  | CsA | 20 | 0.332 | 0.087 | 26.2 | 0.077 | 2.213 | 8.9 | 0.89 | 52.7 |
| BSEP+ATP |  |  |  |  | 2.440 | 0.143 | 5.84 | 2.290 |  |  |  |  |
| BSEP+AMP |  |  | Iruplinalkib | 100 | 0.375 | 0.084 | 22.5 | 0.120 | 3.450 | 13.8 | 1.38 | 26.3 |
| BSEP+ATP |  |  |  |  | 3.720 | 0.576 | 15.5 | 3.570 |  |  |  |  |
| BSEP+AMP |  |  |  | 30 | 0.263 | 0.043 | 16.6 | 0.008 | 4.687 | 18.7 | 1.87 | -0.09 |
| BSEP+ATP |  |  |  |  | 4.845 | 0.473 | 9.76 | 4.695 |  |  |  |  |
| BSEP+AMP |  |  |  | 10 | 0.232 | 0.058 | 25.1 | -0.024 | 5.173 | 20.7 | 2.07 | -10.5 |
| BSEP+ATP |  |  |  |  | 5.300 | 0.310 | 5.84 | 5.150 |  |  |  |  |
| BSEP+AMP |  |  |  | 3 | 0.283 | 0.016 | 5.68 | 0.028 | 5.237 | 20.9 | 2.09 | -11.8 |
| BSEP+ATP |  |  |  |  | 5.415 | 0.325 | 6.00 | 5.265 |  |  |  |  |
| BSEP+AMP |  |  |  | 1 | 0.305 | 0.053 | 17.3 | 0.050 | 5.935 | 23.7 | 2.37 | -26.7 |
| BSEP+ATP |  |  |  |  | 6.135 | 0.704 | 11.5 | 5.985 |  |  |  |  |
| BSEP+AMP |  |  |  | 0.3 | 0.272 | 0.071 | 26.2 | 0.017 | 5.183 | 20.7 | 2.07 | -10.7 |
| BSEP+ATP |  |  |  |  | 5.350 | 0.244 | 4.56 | 5.200 |  |  |  |  |
| BSEP+AMP |  |  |  | 0.1 | 0.397 | 0.026 | 6.51 | 0.142 | 5.873 | 23.5 | 2.35 | -25.4 |
| BSEP+ATP |  |  |  |  | 6.165 | 0.069 | 1.11 | 6.015 |  |  |  |  |

Note: SD, standard deviation; CV, coefficient of variation.

**Supplementary Table 13. The results of iruplinalkib as a MPR2 inhibitor**

| **Membrane capsule and treatment** | **Standard substrate** | | **Reference or examination inhibitors** | | **Substrate transfer volume (pmol)** | | | | | **Uptake transport activity (pmol/mg protein/min)** | **Volume of substrate solution transferred (μL/mg protein/min)** | **Inhibition rate (%)** |
| --- | --- | --- | --- | --- | --- | --- | --- | --- | --- | --- | --- | --- |
|  | **Compound** | **Conc**  **(μM)** | **Compound** | **Conc**  **(μM)** | **Mean** | **SD** | **CV (%)** | **Net value** | **ATP-dependent** |  |  |  |
| NC+AMP | CDCF | 5 |  |  | 0.215 | 0.017 | 8.13 |  |  |  |  |  |
| NC+ATP |  |  |  |  | 0.140 | 0.015 | 10.7 |  |  |  |  |  |
| MRP2+AMP |  |  |  |  | 0.560 | 0.033 | 5.97 | 0.345 | 20.75 | 83.0 | 8.30 |  |
| MRP2+ATP |  |  |  |  | 21.23 | 2.520 | 11.9 | 21.09 |  |  |  |  |
| MRP2+AMP |  |  | CsA | 20 | 4.884 | 0.340 | 6.96 | 4.669 | 7.419 | 29.7 | 2.97 | 64.2 |
| MRP2+ATP |  |  |  |  | 12.23 | 0.794 | 6.49 | 12.09 |  |  |  |  |
| MRP2+AMP |  |  | Iruplinalkib | 100 | 0.567 | 0.026 | 4.57 | 0.352 | 22.27 | 89.1 | 8.91 | -7.3 |
| MRP2+ATP |  |  |  |  | 22.76 | 0.675 | 2.96 | 22.62 |  |  |  |  |
| MRP2+AMP |  |  |  | 30 | 0.451 | 0.024 | 5.29 | 0.237 | 21.33 | 85.3 | 8.53 | -2.8 |
| MRP2+ATP |  |  |  |  | 21.71 | 0.243 | 1.12 | 21.57 |  |  |  |  |
| MRP2+AMP |  |  |  | 10 | 0.483 | 0.047 | 9.74 | 0.268 | 21.53 | 86.1 | 8.61 | -3.8 |
| MRP2+ATP |  |  |  |  | 21.94 | 0.291 | 1.33 | 21.80 |  |  |  |  |
| MRP2+AMP |  |  |  | 3 | 0.503 | 0.042 | 8.41 | 0.288 | 21.61 | 86.4 | 8.64 | -4.2 |
| MRP2+ATP |  |  |  |  | 22.04 | 1.059 | 4.80 | 21.90 |  |  |  |  |
| MRP2+AMP |  |  |  | 1 | 0.554 | 0.026 | 4.76 | 0.339 | 21.68 | 86.7 | 8.67 | -4.5 |
| MRP2+ATP |  |  |  |  | 22.16 | 13.63 | 61.5 | 22.02 |  |  |  |  |
| MRP2+AMP |  |  |  | 0.3 | 0.603 | 0.038 | 6.27 | 0.388 | 22.57 | 90.3 | 9.03 | -8.8 |
| MRP2+ATP |  |  |  |  | 23.10 | 1.158 | 5.01 | 22.96 |  |  |  |  |
| MRP2+AMP |  |  |  | 0.1 | 0.559 | 0.026 | 4.70 | 0.345 | 22.26 | 89.0 | 8.90 | -7.3 |
| MRP2+ATP |  |  |  |  | 22.75 | 0.383 | 1.68 | 22.61 |  |  |  |  |

Note: SD, standard deviation; CV, coefficient of variation.

**Xenograft models establishment**

**LU-01-0015 (HIP1-ALK) and LU-01-0319 patient-derived tumor xenograft (PDX) models**

Both LU-01-0015 and LU-01-0319 cancer tissues are derived from different ALK-positive NSCLC patients. PDX models were established with the patients’ informed consent, and followed the the protocol approved by Wuxi AppTec (Shanghai) Co., Ltd. The results of gene sequencing showed that LU-01-0015 carried HIP1-ALK fusion gene, while LU-01-0319 carried EML4-ALK fusion gene. Patient-derived tumor tissue was segmented into small pieces of approximately 20–30 mm^3^ and transplanted subcutaneously into the right back of mice. When the tumor volume reached 150-200 mm^3^, the mice were randomly divided into corresponding groups (9 mice in each group, female). Administered by gavage once daily for 4 weeks.

**NCI-H3122 (EML4-ALK)** **cell line-derived xenograft model**

Human non-small cell lung cancer cell line NCI-H3122 was purchased from Nanjing Kobai Biotechnology Co., Ltd.

NCI-H3122 cells were re-suspended with 0.9% sodium chloride, and then 6×10^6^ cells in 100 μL was inoculated subcutaneously into the right armpit of nude mice to prepare solid tumor models under sterile conditions. After the tumors grew to 100-300 mm^3^, they were randomly divided into 5 groups according to size: blank control group, brigatinib (5 mg/kg) group, iruplinalkib (2.5 mg/kg) group, iruplinalkib (5 mg/kg) group and iruplinalkib (10 mg/kg) group, with 9 animals in each group. Groups were gavaged once daily with the base dose of drug for 21 days. The blank control group was given the same volume of vehicle (sterile water) injection according to the same protocol, with the dose volume of 100 μL/10 g.


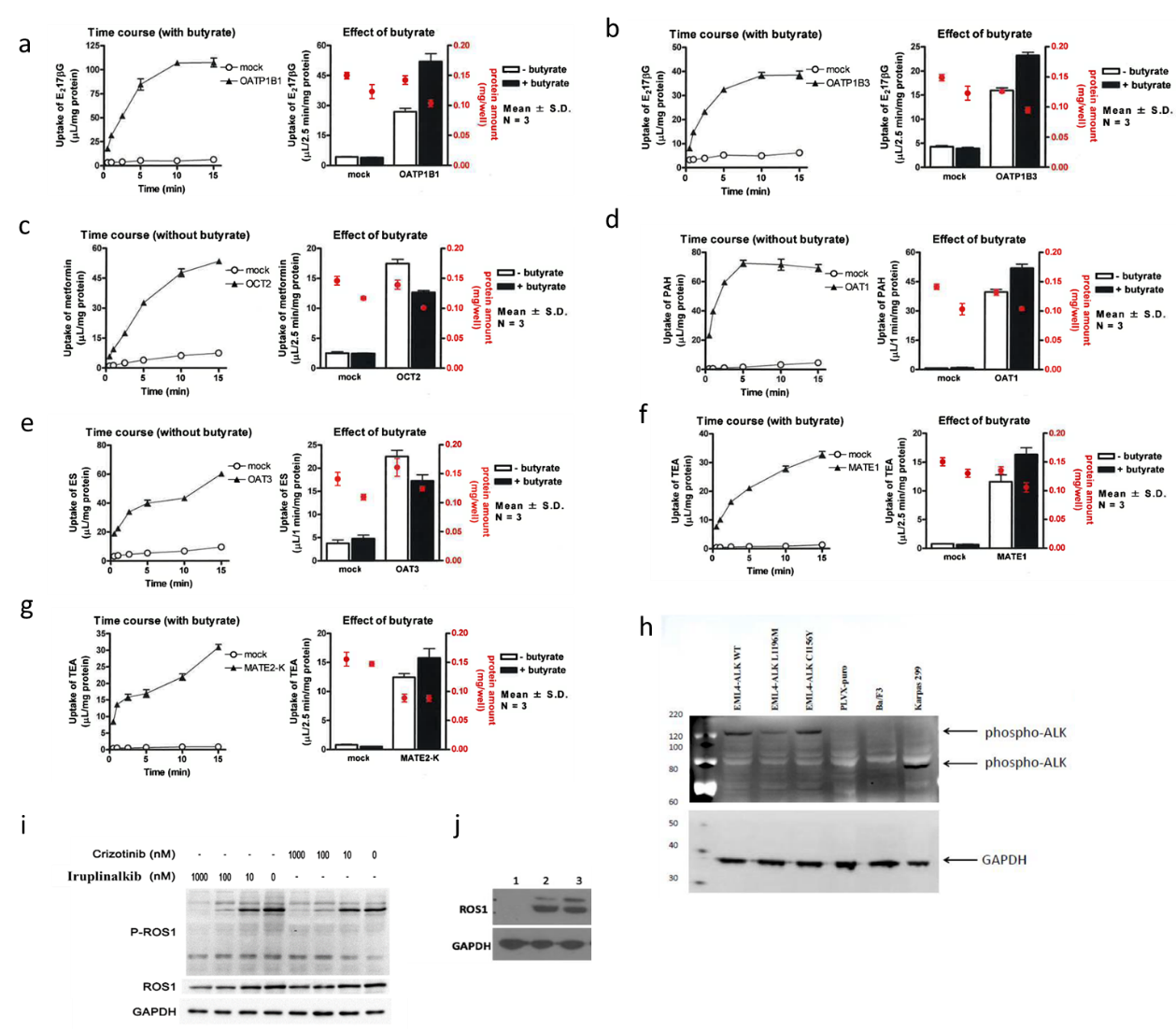


**Supplementary Figure 1.** a-b: The substrate uptake function of *OATP1B1* and *OATP1B3* expressed in HEK293 engineered cell lines were demonstrated by transport activity assay. Substrate: Estradiol 17-β-glucuronide (E217βG). c: The substrate uptake function of *OCT2* expressed in HEK293 engineered cell lines were demonstrated by transport activity assay. Substrate: Metformin. d: The substrate uptake function of *OCT1* expressed in HEK293 engineered cell lines were demonstrated by transport activity assay. Substrate: p-aminohippurate (PAH). e: The substrate uptake function of *OCT3* expressed in HEK293 engineered cell lines were demonstrated by transport activity assay. Substrate: Estrone-3-sulfate (ES). f-g: The substrate uptake function of *MATE1* and *MATE-2K* expressed in HEK293 engineered cell lines were demonstrated by transport activity assay. Substrate: Tetraethylammonium (TEA). h: The levels of phosphorylated ALK in Ba/F3 cell lines transfected with *EML4-ALK WT*, *EML4-ALK L1196M*, and *EML4-ALK C1156Y* after 24 h, and in Karpas 299 cell line (*NPM-ALK* fusion gene) were determined by western blot. i: The levels of ROS1 and phosphorylated ROS1 in engineered NIH-3T3 cell line (*CD74-ROS1* fusion gene) were determined by western blot. j: The levels of ROS1 in Ba/F3 cell line were determined by western blot. Line 1: Ba/F3 cell line; line 2: Ba/F3 *SLC34A2-ROS1* cell line clone 1; line 3: Ba/F3 *SLC34A2-ROS1* cell line clone 2.


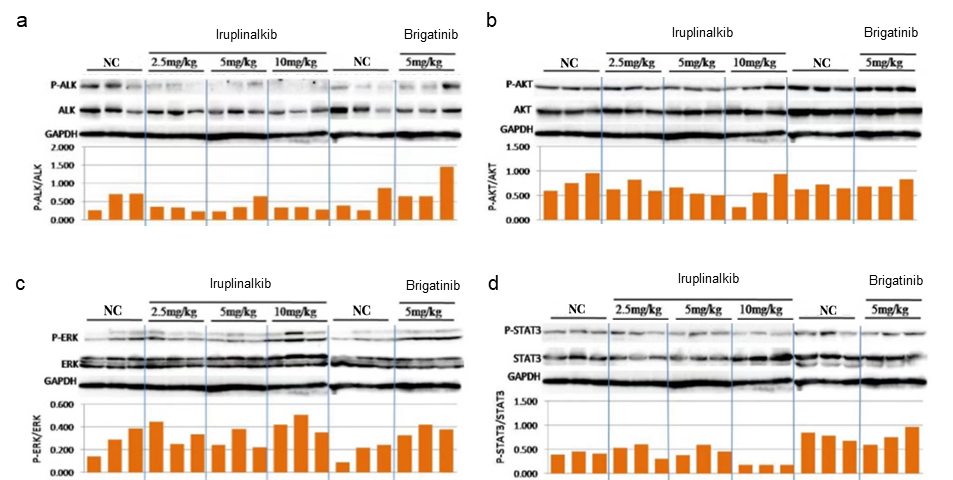


**Supplementary Figure 2.** a-d: the detection of the phosphorylation level of downstream signal molecules in tumor tissue in the NCI-H3122 cell line transplanted tumor (*EML4-ALK*) model 1 h after administration.
